# Machine Learning Reveals Common Regulatory Mechanisms Mediated by Autophagy-Related Genes in the Development of Inflammatory Bowel Disease and Major Depressive Disorder

**DOI:** 10.3390/genes17010004

**Published:** 2025-12-19

**Authors:** Gengxian Wang, Luojin Wu, Jiyuan Shi, Mengmeng Sang, Liming Mao

**Affiliations:** 1 School of Life Science and Engineering, Handan University, Handan 056005, China; wgx123@hdc.edu.cn; 2 Department of Immunology, School of Medicine, Nantong University, Nantong 226001, China; 2431310023@stmail.ntu.edu.cn (L.W.); 2331310021@stmail.ntu.edu.cn (J.S.); 3 Basic Medical Research Center, School of Medicine, Nantong University, Nantong 226019, China

**Keywords:** autophagy, inflammatory bowel disease, major depressive disorder, machine learning algorithm, molecular docking and dynamics analysis

## Abstract

Background: Major Depressive Disorder (MDD) is more common in patients with Inflammatory Bowel Disease (IBD) than in the general population, suggesting a shared but unclear pathogenesis. Autophagy, a conserved intracellular cleaning process, maintains cellular health by removing debris and recycling nutrients. Given the limited research on autophagy in this comorbidity, this study investigated the role of autophagy-related genes in both disorders. Aim: This study aimed to identify shared autophagy-related mechanisms between IBD and MDD and to explore potential therapeutic strategies. Methods: We identified differentially expressed autophagy-related genes (DE-ARGs) in diseased versus normal tissues. Shared DE-ARGs between IBD and MDD were designated Co-DEGs. We analyzed correlations among Co-DEGs and their association with immune cell infiltration. Four machine-learning algorithms were used to pinpoint key biomarkers. Potential therapeutic agents were predicted and validated via molecular docking. Results: We identified 47 shared Co-DEGs. Among these, *CASP1* emerged as a cross-disease shared susceptibility-associated gene (SSAG), consistently selected by all machine-learning models. Drug-gene interaction analysis and molecular docking identified compounds that could regulate *CASP1*. Single-cell analysis suggested *CASP1* helps reshape the immune microenvironment in Crohn’s disease. Furthermore, Mendelian randomization identified WDR6 as a shared genetic risk factor for both conditions. Conclusions: Our findings illuminate autophagy-mediated mechanisms linking gut and brain disorders. The identification of *CASP1* as a SSAG, along with candidate therapeutics, provides a foundation for future research and targeted treatments for IBD and MDD comorbidity.

## 1. Introduction

Inflammatory bowel disease (IBD) is an inclusive term mainly covering two chronic gastrointestinal disorders, Crohn’s disease (CD) and ulcerative colitis (UC). CD is a chronic inflammatory condition affecting the gastrointestinal tract, with its incidence showing a global upward trend. The development of CD is thought to stem from a complex interplay between genetic susceptibility, environmental factors, and altered gut microbiota, which collectively lead to dysregulated innate and adaptive immune responses [1]. Historically, UC has been more prevalent in industrialized Western countries. However, in recent years, its incidence and hospitalization rates have been rising sharply in newly industrialized nations such as China, India, and those in Latin America. By 2023, the global prevalence of UC had reached approximately 5 million [2]. The primary symptoms of UC include bloody diarrhea or rectal bleeding, which affect 90% of patients [2]. Other common symptoms are abdominal pain, fatigue, and increased bowel urgency, all of which significantly diminish patients’ quality of life [3]. It is well documented that in around 25% of IBD cases, the peripheral inflammatory processes are not confined to the intestinal tract alone. These so-called extraintestinal manifestations can impact multiple organs, including the joints, skin, and eyes [4]. In recent times, the connection between IBD and neuropsychiatric disorders, particularly depression and anxiety, has gained widespread recognition [5]. Long-term suffering from IBD increases the risk of developing Major Depressive Disorder (MDD). MDD is a multifactorial psychiatric disorder defined by the presence of at least one depressive episode lasting for a minimum of two weeks. This condition arises from a complex interaction between environmental and genetic factors. Several studies have indicated that gut dysbiosis-induced inflammation may trigger or contribute to the development of depression through the dysregulation of the gut–brain axis [6]. Notably, gut microbial dysbiosis in patients with IBD impairs both the immune system and central nervous system via microbiota-gut crosstalk and gut-brain communication, thereby promoting the onset of anxiety and depression [7,8]. Alterations in the gut microbiota, characterized by shifts in community structure and diminished biodiversity, are established as critical factors in disease pathogenesis. These disruptions compromise intestinal barrier integrity and can drive pathological immune responses in genetically susceptible individuals [9]. Regarding therapeutic interventions, microbiota-based treatments, such as the transfer of processed donor fecal material to patients with mild-to-moderate UC, have demonstrated promise. Early-phase clinical trials report that approximately 30% of participants achieved remission following this therapy [10].

Autophagy is the process by which cells degrade and recycle proteins and organelles to maintain intracellular homeostasis. Generally, autophagy plays a protective role in cells, but disruption of autophagy mechanisms or excessive autophagic flux usually leads to cell death [11]. Autophagy, commonly referring to macro-autophagy, is a conserved cellular degradation process. During this process, cytoplasmic components are sequestered within double-membraned vesicles termed autophagosomes, which subsequently fuse with lysosomes for cargo breakdown and recycling [12]. A compromised intestinal barrier is a hallmark of IBD. Intestinal epithelial cells (IECs), as crucial constituents of this mucosal barrier, not only regulate paracellular permeability but also serve as the primary line of defense against luminal pathogens [13]. In patients with CD, A compromised intestinal barrier is a hallmark of IBD. IECs, as crucial constituents of this mucosal barrier, not only regulate paracellular permeability but also serve as the primary line of defense against luminal pathogens [14]. In the UC mouse model, treatment with licorice extract alleviated histopathological damage, lowered pro-inflammatory cytokines (IL-6, IL-17), and mitigated mitochondrial impairment. These protective effects are potentially mediated through the Nrf2/PINK1 signaling pathway, which is known to modulate autophagy [15]. Furthermore, emerging evidence highlights a critical crosstalk between autophagy pathways and the NLRP3 inflammasome, offering a promising immunological perspective for developing novel therapeutic strategies in depression [16]. Furthermore, a recent investigation discovered autophagy-related hub genes that are involved in regulating the immune microenvironment in MDD, using bioinformatics analyses and experimental validation [17]. Nevertheless, to date, no research has identified ARGs that exert simultaneous effects on both IBD and MDD, nor have any therapeutics been found to effectively target ARGs for modulating the progression of these two diseases.

To elucidate the shared autophagy-mediated mechanisms linking IBD and MDD, we conducted a comprehensive bioinformatics study. In this study, we systematically integrated publicly available transcriptomic datasets across intestinal tissues (CD [18,19,20] and UC [19,20,21]) and central or peripheral nervous system tissues from MDD [22,23,24]. Autophagy-related genes were curated primarily from Zou et al. and represent a widely recognized reference gene set for autophagy-mediated regulatory pathways (Supplementary Table S1) [18]. To identify cross-disease autophagy mechanisms, we defined co-differentially expressed genes (Co-DEGs) as ARGs consistently dysregulated in both IBD and MDD across tissues. This concept allows the detection of shared pathological signatures underlying gut–brain axis dysfunction.

Our analysis not only identified *CASP1* as a cross-disease SSAG between the two disorders but also delineated its central role in orchestrating immune-inflammatory responses across the gut-brain axis. By integrating multiple machine-learning algorithms, we established *CASP1* as a robust common SSAG. Moving beyond target identification, we leveraged drug-gene interaction databases and molecular docking to predict several candidate drugs capable of modulating *CASP1* activity, thereby offering a tangible strategy for dual-purpose therapy. These findings decipher a novel pathogenic link and provide a foundational framework for understanding the comorbidity, ultimately paving the way for mechanism-based therapeutics for patients suffering from both conditions.

## 2. Materials and Methods

### 2.1. Data Source

The datasets were all retrieved from the GEO database, with samples derived from colon biopsies in both the CD and UC datasets. The datasets employed for CD included GSE20881 (comprising 73 control samples and 99 CD samples) [19], GSE24287 (comprising 25 control samples and 47 CD samples) [20], and GSE179285 (comprising 31 control samples and 168 CD samples) [21]. For UC, the utilized datasets were GSE13367 (including 20 control samples and 34 UC samples) [22], GSE24287 (including 25 control samples and 27 UC samples) [20], and GSE179285 (including 31 control samples and 55 UC samples) [21]. Two datasets were used for MDD: GSE98793 for peripheral blood samples (containing 64 control samples and 128 MDD samples [23], and GSE19738 for whole blood samples (containing 66 control samples and 66 MDD samples) [24]. Additionally, MDD-related datasets included GSE54568 from the prefrontal cortex of the human brain, GSE54571 from the anterior cingulate cortex of the human brain, and GSE54564 from the amygdala tissue of the human brain. Specifically, the GSE54568 dataset included 15 control samples and 15 MDD samples [25], the GSE54571 dataset included 13 control samples and 13 MDD samples [25], and the GSE54564 dataset included 21 control samples and 21 MDD samples [25]. We merged the three CD datasets, three UC datasets, and two MDD whole blood datasets separately. Batch effects across these datasets were removed by implementing the Com Bat algorithm in the sva R package [26] (v3.52.0).

### 2.2. Identification of DE-ARGs and Immune Cell Infiltrations

DE-ARGs were identified by comparing healthy controls with affected individuals across CD, UC, and the various MDD tissue cohorts. Differential expression of autophagy-related genes (ARGs) was assessed using the Wilcoxon rank-sum test. For each dataset, we report both the nominal *p*-value and the Benjamini–Hochberg FDR–adjusted q-value. Given the modest sample sizes and our hypothesis-driven focus on a predefined ARG set, we applied a nominal threshold of *p* < 0.05 to define exploratory DE-ARGs and Co-DEGs for downstream analyses, and we interpret these findings as hypothesis-generating rather than confirmatory. The resulting gene expression profiles were then graphically represented in the form of heatmaps using the pheatmap software package (v1.0.13) [27]. Common differentially expressed autophagy-related genes (Co-DEGs)—defined as those consistently dysregulated in both IBD and each independent MDD tissue dataset—were subsequently extracted. To evaluate the potential relationships among these overlapping genes, pairwise Pearson correlation analysis was conducted. The resulting correlation matrix was then graphically represented using the corrplot package (version 0.95) [28].

### 2.3. Immune Cell Infiltrations

To characterize immune microenvironmental differences between disease and control samples, we quantified immune cell infiltration using the CIBERSORT algorithm implemented in the IOBR package (v0.99.9). CIBERSORT deconvolves bulk RNA-seq expression matrices into the relative proportions of 22 immune cell subsets. Group-wise comparisons of immune-cell fractions between disease and control tissues were performed using non-parametric tests. To explore whether Co-DEGs were associated with immune alterations, Pearson correlation coefficients were computed between the expression levels of the 47 Co-DEGs and the estimated proportions of each immune cell type. Correlation matrices and individual gene–immune associations were visualized using the ggplot2 package (v3.5.1) [29], enabling identification of immune-cell populations most strongly influenced by autophagy-related transcriptional dysregulation.

### 2.4. Machine Learning-Based Screening of Potential Biomarkers

Machine-learning–based screening of potential biomarkers was performed to identify Co-DEGs with the strongest discriminatory power across disease states. Four complementary algorithms—Support Vector Machine (SVM) [29], Random Forest (RF) [30], Generalized Linear Model (GLM) [31], and eXtreme Gradient Boosting (XGB) [32]—were implemented to ensure robustness and reduce model-specific bias. Prior to model training, all gene-expression features were standardized using z-score normalization, and each dataset was randomly partitioned into training and test sets using a 70:30 split. All models were trained using default hyperparameter settings implemented in the respective R packages (caret (v 7.0.1), randomForest (v 4.7.1.2), glmnet (v 4.1.10), and xgboost (v 1.7.11.1)). To reduce overfitting and ensure model stability, 10-fold cross-validation was performed within the training set for each algorithm. Each algorithm then independently evaluated the contribution of each Co-DEG to classifying normal versus disease samples. Class distributions across disease and control groups were relatively balanced; therefore, no additional resampling was required. For each method, genes were ranked according to their feature-importance scores, and the top 20 genes were selected as high-value predictors. To minimize algorithm-specific variance and identify the most reproducible markers, we intersected the top-ranked gene sets from all four methods. Genes consistently selected across algorithms were defined as robust biomarkers associated with disease occurrence. Model performance was further assessed by generating receiver-operating characteristic (ROC) curves and calculating standard classification metrics, including area under the curve (AUC), accuracy, precision, recall, and F1-score. These complementary evaluation indices enabled a comprehensive assessment of each algorithm’s discriminatory capability and ensured the robustness and reliability of the selected biomarkers.

### 2.5. Differential Analysis and Gene Set Variation Analysis (GSVA) Between Subgroups

Samples were stratified into *CASP1*^+^ and *CASP1*^−^ groups based on the median *CASP1* expression level within each dataset to investigate the biological effects associated with *CASP1* dysregulation. Differential expression analysis between the two groups was performed using the limma–voom framework, and gene-level changes were visualized as volcano plots generated with the ggplot2 package (v3.5.1). To further characterize functional alterations linked to *CASP1* expression, we applied Gene Set Variation Analysis (GSVA), a non-parametric and unsupervised approach that estimates pathway-level enrichment from sample-wise expression profiles. KEGG pathway gene sets were retrieved from the MSigDB database, and GSVA enrichment scores were calculated for each sample to identify shifts in pathway activity between *CASP1*^+^ and *CASP1*^−^ groups [30].

### 2.6. Drug Prediction

To identify chemical compounds potentially targeting *CASP1* dysregulation, we retrieved z-score–normalized half-maximal inhibitory concentration (IC50) values for all compounds, together with log_2_ (FPKM + 1)–normalized RNA-seq profiles of the NCI-60 panel of human cancer cell lines from the CellMiner database (https://discover.nci.nih.gov/cellminer/, accessed on 18 November 2025). Pearson correlation analyses were performed to quantify the association between *CASP1* transcript abundance and compound activity z-scores across the 60 cell lines. Compounds exhibiting significant correlations (*p* < 0.01) were considered putative agents that may positively or negatively modulate *CASP1*-associated biological processes.

### 2.7. Molecular Docking Analysis

The molecular docking model was prepared using a protein structure obtained from the Protein Data Bank (https://www.uniprot.org/), from which any bound ligands were subsequently removed. Corresponding small-molecule drug structures were sourced from two public databases: ZINC15 (https://wiki.docking.org/index.php/) and the PubChem Small Molecule Database (https://pubchem.ncbi.nlm.nih.gov/). Molecular docking and receptor-drug visualization were conducted with PyMol software (v3.0.5), and binding free energies were computed in turn.

### 2.8. scRNA-seq Analysis

In this study, we analyzed the single-cell RNA-seq dataset GSE214695, which comprises 12 colon samples from patients with CD. A total of 46,700 cells passed initial quality control and were processed using the Seurat R package (v5.3.0). All data from the collected samples were combined into a single Seurat object. Following this integration, cells of low quality were excluded according to established thresholds for both mitochondrial gene content and the total number of genes detected per cell. We then performed principal component analysis (PCA) to evaluate major sources of variation and determine the optimal number of principal components for downstream analyses. UMAP was subsequently used for dimensionality reduction and visualization. Major cell types were annotated using the SingleR package (v2.4.1). Within each cell type, cells were stratified into *CASP1*^+^ and *CASP1*^−^ subpopulations using a *CASP1* expression cutoff of zero. The FindMarkers function was employed to identify DEGs, with significance thresholds set at *p* < 0.05 and |log_2_ fold-change| > 1. To explore intercellular communication, we used the CellChat package (v1.6.1) to infer signaling interactions, integrating ligand–receptor pairs along with essential cofactors to construct a comprehensive communication network.

### 2.9. Mendelian Randomization Analysis

We employed the TwoSampleMR R package (v0.6.18) to evaluate the causal effects of Co-DEGs on CD, UC, and MDD, treating Co-DEGs as exposures and the three diseases as outcomes. For CD, three genome-wide association studies (GWAS) were included (ebi-a-10, ieu-a-12, ieu-a-30), and for UC, three GWAS datasets were used (ebi-a-32, ieu-a-970, ieu-a-973). In addition, twelve MDD GWAS datasets (ieu-a-1188, ieu-a-GCST005903, ieu-a-GCST005904, ieu-a-GCST0086061, ieu-a-GCST009981, ieu-a-GCST009983, ieu-a-GCST009984, ieu-a-GCST0086058, ieu-a-GCST0086059, ieu-a-GCST0086060, ieu-a-GCST0086061, ieu-a-GCST0086062) were retrieved from the IEU OpenGWAS database (https://gwas.mrcieu.ac.uk/). Independent SNPs associated with each Co-DEG at *p* < 5 × 10^−4^ were selected as instrumental variables (IVs). To ensure independence, SNPs were clumped using an LD threshold of *r^2^* < 0.001 within a 10,000-kb window. Instrument strength was assessed using F-statistics, and SNPs with F < 10 were excluded to minimize weak-instrument bias. Horizontal pleiotropy was evaluated using MR-Egger regression, and heterogeneity among instruments was assessed via Cochran’s Q statistic. The inverse-variance weighted (IVW) method and MR-Egger regression were used as the primary estimators of causal effects. When significant directional pleiotropy was detected, the MR-Egger estimate was prioritized; otherwise, the IVW estimate was taken as the main result. For IVW analyses, a random-effects model was applied when Cochran’s Q test indicated heterogeneity (*p* < 0.05), and a fixed-effects model was used otherwise. Leave-one-out analyses were additionally performed to assess whether any single SNP disproportionately influenced the causal estimates.

### 2.10. Statistical Analysis

All statistical analyses were performed using R software (v4.4.2). For comparisons between two groups, the Wilcoxon rank-sum test was applied. Correlation analyses were conducted using Pearson correlation coefficients. Unless otherwise specified, statistical significance was defined as *p* < 0.05.

## 3. Results

### 3.1. Detection of Co-DEGs Between IBD and MDD

**Figure 1:**
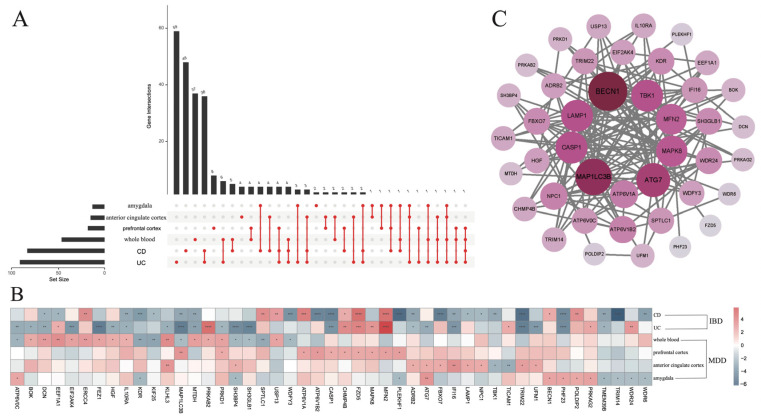
Identification of Co-DEGs associated with CD, UC, and MDD. (**A**) Upset plot illustrating the distribution of DEGs in CD, UC, and MDD. (**B**) Heatmap of the expression profiles of 47 Co-DEGs in CD, UC, whole blood, prefrontal cortex, anterior cingulate cortex, and amygdala samples. (**C**) The protein-protein interaction network of 47 Co-DEGs. *: *p* < 0.05; **: *p* < 0.01; ***: *p* < 0.001; ****: *p* < 0.0001.

This investigation aimed to examine the potential role of autophagy as a common pathological mechanism underlying both inflammatory bowel disease (IBD) and major depressive disorder (MDD), by identifying and analyzing differentially expressed genes (DEGs) shared between these two conditions. To address the critical knowledge gap surrounding the molecular basis of IBD-MDD comorbidity, this study pivots from a singular disease focus to a mechanism-centered inquiry, positioning autophagy as a putative linchpin. We hypothesized that a comparative transcriptomic analysis across gut and brain tissues would unveil a conserved, autophagy-mediated pathogenic program. Gene expression data were obtained from the GEO database, encompassing samples of CD colon, UC colon, MDD whole blood, along with MDD prefrontal cortex, anterior cingulate cortex, and amygdala. We first merged GSE20881, GSE24287, and GSE179285, which include control and CD samples, to construct a combined control–CD dataset. Similarly, GSE13367, GSE24287, and GSE179285 were merged to generate a unified control–UC dataset. For MDD, GSE98793 and GSE19738 containing control and peripheral blood MDD samples were integrated to create a consolidated control–MDD peripheral blood dataset (Supplementary Figure S1). For the 368 ARGs, we identified 126 DE-ARGs in UC, 115 in CD, 64 in MDD whole blood, 25 in the MDD prefrontal cortex, 21 in the MDD anterior cingulate cortex, and 18 in the MDD amygdala (Figure 1A). Among these, 47 ARGs were consistently dysregulated in each IBD subtype (CD and UC) and each independent MDD tissue dataset; we therefore defined these 47 genes as Co-DEGs. Both nominal *p*-values and FDR-adjusted q-values for these 47 genes are provided in Supplementary Table S2, and all DEG findings are interpreted as exploratory signals that motivate further validation. To elucidate the functional relationships among these overlapping genes, a protein-protein interaction (PPI) network was constructed using the STRING database. This network analysis highlighted several central hub proteins, including *BECN1*, *TBK1*, *MFN2*, *MAPK8*, *ATG7*, *ATP6V1A*, *MAP1LC3B*, *CASP1*, and *LAMP1* (Figure 1C).

### 3.2. Correlation Analysis of the 47 Co-DEGs

To elucidate the regulatory interplay of the 47 overlapping differentially expressed genes under both physiological and pathological states, we performed detailed co-expression analyses stratified by individual tissue groups. The correlation matrices for both control and disease samples are presented in Supplementary Figure S2A–F. In the presented visualizations, control samples are positioned within the lower-left quadrant, whereas disease samples are located in the upper-right quadrant. The correlation patterns of the 47 overlapping differentially expressed genes exhibited notable tissue-specific heterogeneity. Notably, the prefrontal cortex and anterior cingulate cortex exhibited markedly tighter linear relationships among these genes, in both normal and MDD samples, compared with other tissues (Supplementary Figure S2C,D). In addition, the linear associations among the 47 Co-DEGs differed substantially between normal and disease states. For example, the negative correlations of *CHMP4B* with *BOK*, *DCN*, *FEZ1*, *HGF*, *IL10RA*, and *KDR* were considerably stronger in CD than in normal samples (Supplementary Figure S2B), suggesting disease-specific rewiring of Co-DEG interactions. Overall, these analyses reveal that the interaction patterns among the 47 Co-DEGs vary substantially across tissues and disease states, reflecting a pronounced disease-specific rewiring of their regulatory relationships.

### 3.3. The Differential Infiltration of Immune Cells

To explore whether shared autophagy-related signatures correspond to alterations in the immune microenvironment, we examined the association between common autophagy-related gene expression and changes in immune cell composition. Using the CIBERSORT algorithm to characterize differences in immune cell composition. Plasma cells, resting NK cells, and neutrophils showed significantly higher infiltration in both CD and UC tissues compared with normal controls (Table 1). In MDD, only M0 macrophages exhibited significantly elevated infiltration in blood samples relative to normal subjects (Table 2). In contrast, we observed no evidence for altered immune cell infiltration in MDD within the examined brain regions: the prefrontal cortex, anterior cingulate cortex, and anterior amygdala (Table 2).

### 3.4. Correlation Analysis Between 47 Co-DEGs and Immune Cells

To further determine whether the shared autophagy-related signatures influence immune dysregulation, We examined the associations between the 47 common differentially expressed genes (Co-DEGs) and immune cell infiltration in specimens from CD colon, UC colon, MDD whole blood, as well as MDD prefrontal cortex, anterior cingulate cortex, and amygdala (Supplementary Figure S3A–F). Across both IBD and MDD samples, several immune cell subtypes showed significant associations with the expression patterns of these Co-DEGs. For instance, a positive correlation was observed between Macrophages M0 and KDR expression in both CD colon and MDD whole blood samples. These results imply that the identified Co-DEGs might influence the recruitment or abundance of particular immune cell populations through modulatory interactions, potentially playing a role in the disease mechanisms of IBD and MDD.

### 3.5. Machine Learning Screening for the Important Genes

**Figure 2:**
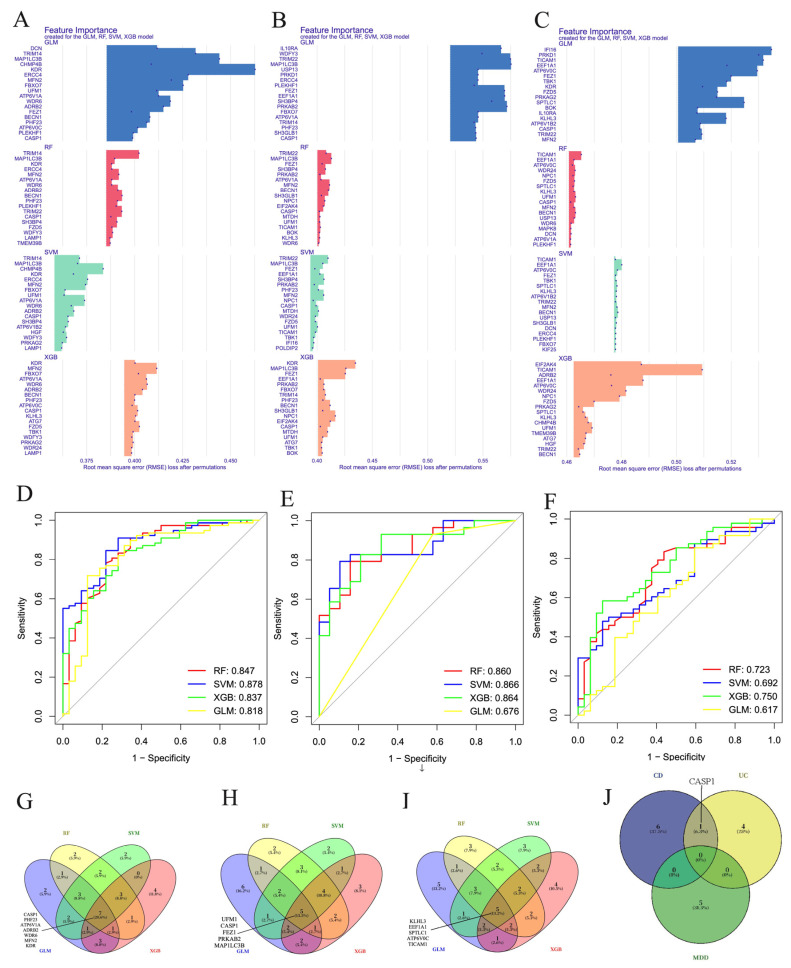
Machine-learning identification of key Co-DEGs associated with CD, UC, and MDD. The top 20 Co-DEGs for (**A**) CD colon samples, (**B**) UC colon samples, and (**C**) MDD whole blood samples were identified using GLM, RF, SVM, and XGB. (**D**–**F**) ROC curves of CD, UC and MDD. Venn diagrams showing the overlapping key genes detected by the four algorithms in (**G**) CD colon, (**H**) UC colon, and (**I**) MDD whole blood. (**J**) Venn diagram illustrating the shared genes across CD, UC, and MDD.

To identify the most influential ARGs driving disease susceptibility, we next applied multiple machine-learning algorithms to prioritize Co-DEGs with the highest discriminatory power across IBD and MDD datasets. Given the small cohort sizes for MDD prefrontal cortex, anterior cingulate cortex, and amygdala specimens, the predictive performance of machine learning models was consequently limited. We therefore focused subsequent modeling efforts on colon tissues from patients with CD or UC, along with whole blood samples from MDD individuals. Using support vector machine (SVM), random forest (RF), extreme gradient boosting (XGB), and generalized linear model (GLM) algorithms, we identified the twenty most prominent Co-DEGs for CD (Figure 2A), UC (Figure 2B), and MDD whole blood (Figure 2C). We then assessed the diagnostic performance of the models by constructing Receiver Operating Characteristic (ROC) curves and calculating the Area Under the Curve (AUC). For CD, the AUC values were 0.847, 0.878, 0.837, and 0.818 for RF, SVM, XGBoost, and GLM, respectively (Figure 2D). For UC, the AUCs were 0.860, 0.866, 0.864, and 0.676 for RF, SVM, XGBoost, and GLM, respectively (Figure 2E). For MDD whole blood, the AUC values were 0.723, 0.692, 0.750, and 0.617 for RF, SVM, XGBoost, and GLM, respectively (Figure 2F). Consistent with the AUC results, the additional diagnostic metrics—including accuracy, precision, recall, and F1-score—further supported the performance differences among models (Supplementary Table S3). For CD, SVM and GLM achieved the highest overall performance, with accuracy values of 0.836 and 0.836, F1-scores of 0.885 and 0.889, and consistently high precision and recall. For UC, XGBoost demonstrated the strongest performance, showing the highest accuracy (0.833), precision (0.818), and F1-score (0.871). For MDD whole blood, XGBoost again outperformed the other models, with the highest accuracy (0.700) and F1-score (0.774), whereas SVM yielded the highest recall (0.958). Together, these metrics provide a more comprehensive confirmation that SVM performs best for CD, XGBoost performs best for UC and MDD blood, and that the selected Co-DEGs exhibit robust diagnostic value across diseases. *CASP1*, *PHF23*, *ATP6V1A*, *ADRB2*, *WDR6*, *MFN2*, and *KDR* were identified as key biomarkers for CD based on the intersection of the top 20 genes selected by each machine-learning algorithm (Figure 2G). Similarly, *UFM1*, *CASP1*, *FEZ1*, *PEKAB2*, and *MAP1LC3B* were identified as key biomarkers for UC (Figure 2H), while *KLHL3*, *EEF1A1*, *SPTLC1*, *ATP6V0C*, and *TICAM1* were identified as key biomarkers for MDD whole-blood samples (Figure 2I). Notably, *CASP1* emerged as a SSAG between CD and UC (Figure 2J).

### 3.6. Comparison and Analysis of CASP1^+^ and CASP1^−^ Groups

*CASP1* was identified as a key disease-associated gene for both CD and UC. *CASP1* expression was downregulated in CD and UC samples but upregulated in MDD prefrontal cortex samples compared with normal controls. Based on *CASP1* RNA expression levels, we stratified CD, UC, and MDD prefrontal cortex samples into *CASP1*^+^ and *CASP1*^−^ groups using the median *CASP1* expression value as the cutoff.

**Figure 3:**
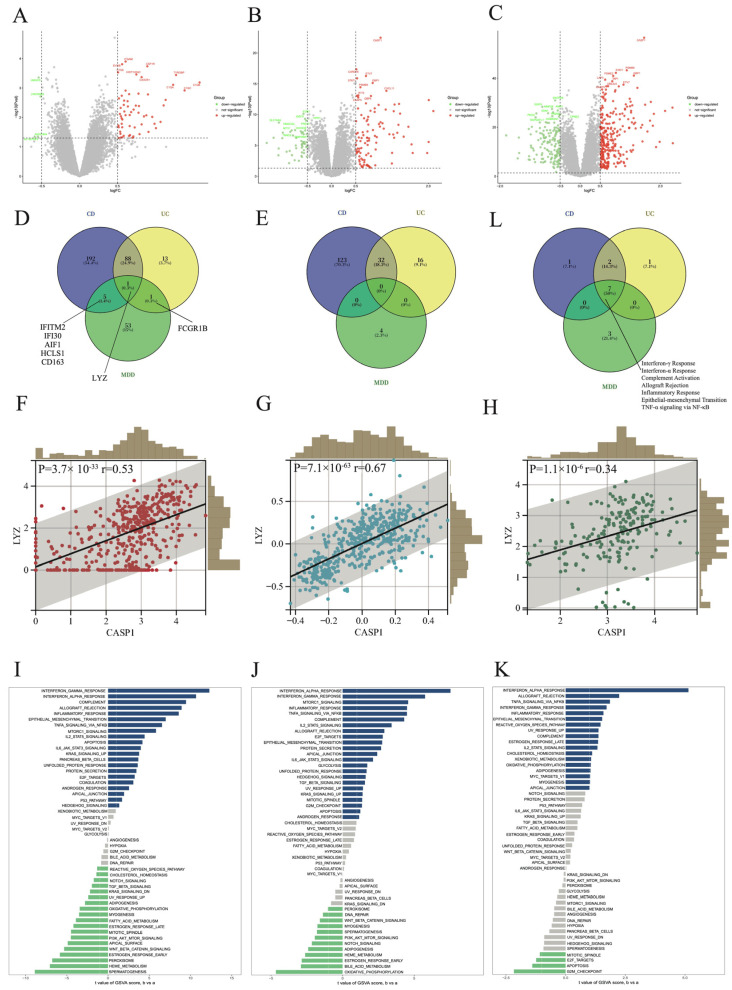
Differential expression and KEGG pathway analyses between *CASP1*^+^ and *CASP1*^−^ groups in CD, UC, and MDD prefrontal cortex. (**A**–**C**) Volcano plots showing DEGs between *CASP1*^+^ and *CASP1*^−^ groups in (**A**) CD, (**B**) UC, and (**C**) MDD prefrontal cortex. (**D**,**E**) Venn diagrams illustrating the overlap of (**D**) upregulated and (**E**) downregulated DEGs among CD, UC, and MDD prefrontal cortex. (**F**–**H**) Correlations between *CASP1* and LYZ RNA expression in (**F**) CD, (**G**) UC, and (**H**) MDD prefrontal cortex. (**I**–**K**) KEGG pathways differentially enriched between *CASP1*^+^ and *CASP1*^−^ groups in (**I**) CD, (**J**) UC, and (**K**) MDD prefrontal cortex. (**L**) Venn diagram showing the overlap of *CASP1*^+^–enriched KEGG pathways across CD, UC, and MDD prefrontal cortex.

In *CASP1*^+^ samples, 286 upregulated and 155 downregulated genes were identified in CD (Figure 3A), 103 upregulated and 48 downregulated genes in UC (Figure 3B), and 60 upregulated and 4 downregulated genes in MDD prefrontal cortex (Figure 3C). LYZ was identified as a shared upregulated gene across all three disease groups (Figure 3D,E), and *CASP1* expression showed a significant positive correlation with LYZ in each dataset (Figure 3F–H).

GSVA further revealed distinct pathway signatures between *CASP1*^+^ and *CASP1*^−^ groups. In CD, samples characterized by elevated *CASP1*^+^ expression were predominantly associated with enrichment in interferon-γ response, interferon-α response, and complement pathways, whereas the *CASP1*^−^ group was enriched in spermatogenesis, heme metabolism, and peroxisome pathways (Figure 3I). In UC, the *CASP1*^+^ group was enriched in interferon-α response, interferon-γ response, and mTORC1 signaling, while the *CASP1*^−^ group was enriched in oxidative phosphorylation and bile acid metabolism (Figure 3J). In MDD prefrontal cortex samples, the *CASP1*^+^ group showed enrichment in interferon-α response, allograft rejection, and TNF-α signaling via NF-κB, whereas the *CASP1*^−^ group was enriched in G2M checkpoint, apoptosis, and E2F targets (Figure 3K).

Notably, interferon-γ response, interferon-α response, complement activation, allograft rejection, inflammatory response, epithelial–mesenchymal transition, and TNF-α signaling via NF-κB were consistently upregulated in the *CASP1*^+^ group across all three disease contexts (Figure 3L).

### 3.7. Drug Prediction and Molecular Docking Analysis

**Figure 4:**
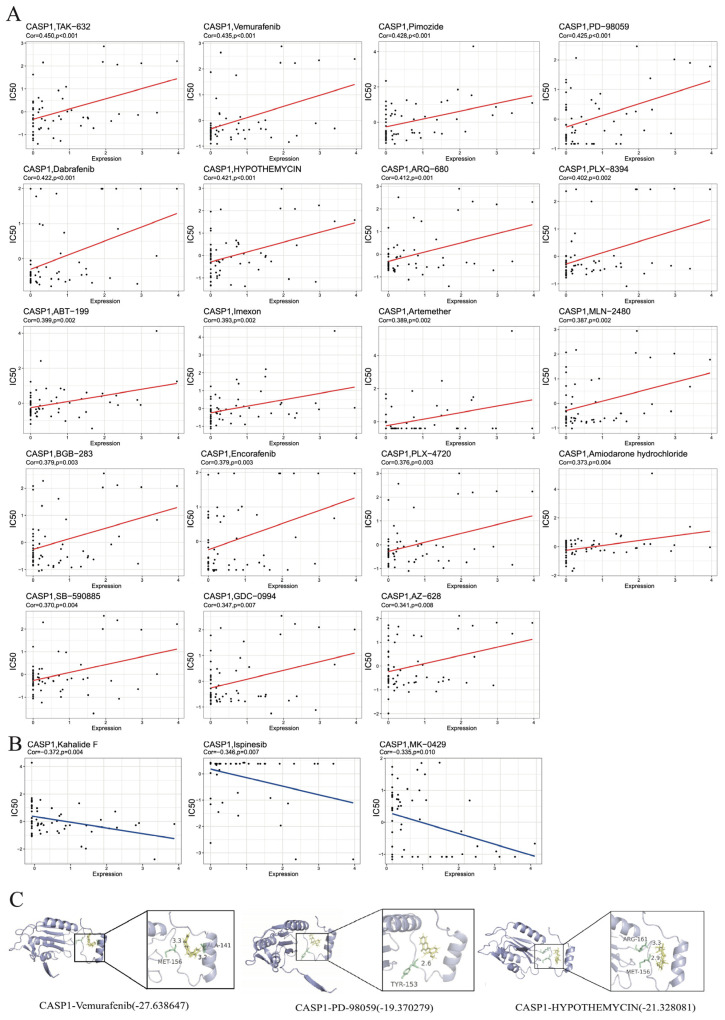
Drug prediction and molecular docking analysis. (**A**) Screening of 19 chemical compounds positively correlated with *CASP1* expression, based on the association between drug IC50 values and gene expression. (**B**) Identification of three compounds showing significant negative correlation with ISCA1 expression. (**C**) Molecular docking analysis of *CASP1* with Vemurafenib, PD-98059, and HYPOTHEMYCIN.

To explore the potential therapeutic relevance of *CASP1* dysregulation, we next integrated drug sensitivity profiling with molecular docking to identify compounds capable of modulating *CASP1* activity. Utilizing the correlation patterns observed among the pharmacological agents IC50 values and *CASP1* RNA expression, we identified 19 chemical compounds that were positively associated with higher *CASP1* expression (Figure 4A), whereas only three compounds showed significant negative correlations with *CASP1* expression (Figure 4B).

All candidate drugs are FDA-approved, having completed the requisite clinical trials. From these, we selected the five strongest positively correlated drugs and the three strongest negatively correlated drugs for molecular docking analysis. However, only the chemical structures of Vemurafenib, PD-98059, and HYPOTHEMYCIN were available for download from the ZINC15 database. Therefore, these three compounds were prioritized for further investigation through molecular docking against the complete *CASP1* structure to evaluate their potential for binding. The resulting docking scores for Vemurafenib, PD-98059, and HYPOTHEMYCIN were −27.64, −19.37, and −21.33, respectively, indicating favorable binding affinities (Figure 4C). Vemurafenib formed hydrogen bonds with ALA-141 and MET-156 of *CASP1*, PD-98059 interacted with *CASP1* via a hydrogen bond at TYR-153, and HYPOTHEMYCIN formed hydrogen bonds with ARG-161 and MET-156 (Figure 4C). These interactions highlight the potential of these compounds to modulate *CASP1* activity through direct binding.

### 3.8. ScRNA-seq Analysis Revealed That CASP1 Regulates the Immune Microenvironment and Cell–Cell Communication in CD

**Figure 5:**
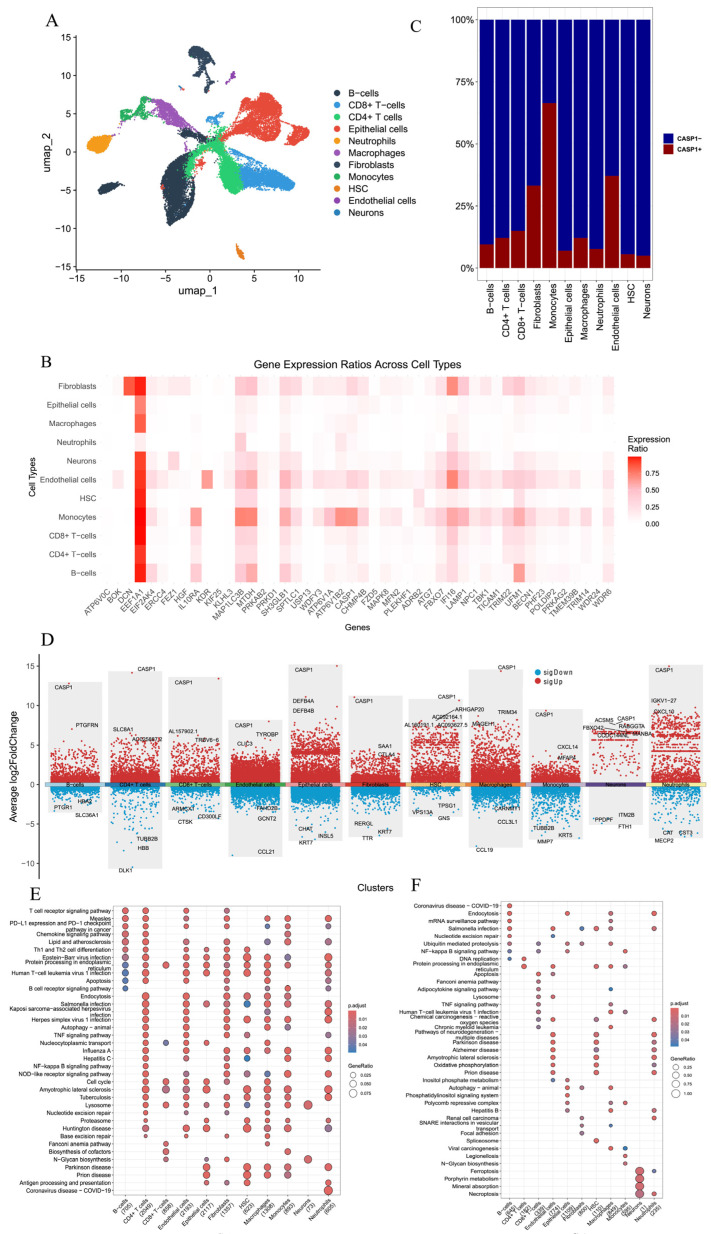
Single-cell sequencing analysis of *CASP1* in CD. (**A**) Cell-type annotation. (**B**) Heatmap showing the distribution of the 47 DE-FRGs across cell types. (**C**) Bar plot illustrating the proportions of *CASP1*^+^ and *CASP1*^−^ cells within each cell type. (**D**) Scatter plot of differential expression analysis between *CASP1*^+^ and *CASP1*^−^ cells. (**E**,**F**) KEGG pathway enrichment of (**E**) upregulated DEGs and (**F**) downregulated DEGs.

To validate the bulk-level findings at single-cell resolution and to elucidate how *CASP1* shapes cellular heterogeneity within the intestinal microenvironment, we next performed scRNA-seq analyses using the GSE214695 dataset. First, the 1500 genes with the highest cell-to-cell variability were selected for downstream analysis, followed by PCA for dimensionality reduction. Cells were subsequently clustered using UMAP and annotated into distinct populations (Figure 5A). The expression patterns of the 47 Co-DEGs were then examined across these cell types (Figure 5B), revealing that EEF1A1 was broadly expressed in most populations except neutrophils, whereas *CASP1* expression was predominantly restricted to monocytes.

We then analyzed the prevalence of *CASP1*^+^ and *CASP1*^−^ cells within each of the 11 designated cell types based on *CASP1* expression (Figure 5C). *CASP1*^−^ cells were particularly enriched within neutrophils. Differential expression analysis was then conducted between *CASP1*^+^ and *CASP1*^−^ cells (Figure 5D). The resulting upregulated and downregulated genes were subjected to KEGG enrichment analysis, highlighting several shared pathways, including apoptosis, endoplasmic reticulum–associated processes, Salmonella infection, NF-κB signaling, Fanconi anemia, amyotrophic lateral sclerosis, Parkinson’s disease, HTLV-1 infection, lysosome, TNF signaling, endocytosis, autophagy (animal), and N-glycan biosynthesis (Figure 5E,F).

**Figure 6:**
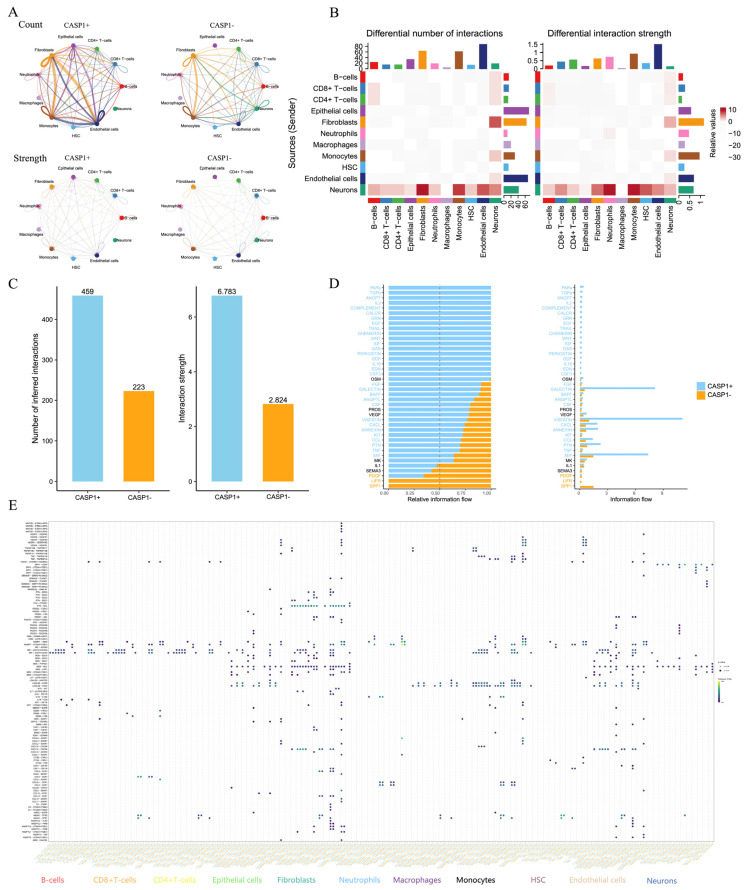
Cell–cell communication analysis between *CASP1*^+^ and *CASP1*^−^ cells. (**A**) Overall number and strength of intercellular interactions among different cell types in *CASP1*^+^ and *CASP1*^−^ groups. (**B**) Differential interaction patterns across cell types. (**C**) Bar plot comparing the inferred interaction numbers and interaction strengths between *CASP1*^+^ and *CASP1*^−^ cells. (**D**) Visualization of differences in molecular information flow between *CASP1*^+^ and *CASP1*^−^ groups. (**E**) Bubble plot illustrating ligand–receptor–mediated cell–cell communication.

To evaluate the impact of *CASP1* expression on intercellular signaling, we compared cell–cell communication networks between *CASP1*^+^ and *CASP1*^−^ conditions (Figure 6A). The analysis of both interaction number and interaction strength revealed that *CASP1* expression markedly reshaped communication patterns within the immune microenvironment. Endothelial cells exhibited the greatest alteration in both interaction count and interaction intensity, while fibroblast–epithelial interactions also showed notable modulation (Figure 6B). Overall, *CASP1*^+^ cells displayed substantially enhanced communication activity compared with *CASP1*^−^ cells (Figure 6C).

Cytokine-related information flow further highlighted distinct communication signatures between *CASP1*^+^ and *CASP1*^−^ states (Figure 6D). Relative information flow analysis indicated that PDGF, VEGF, and MIF contributed more prominently to signaling in the *CASP1*^−^ group, whereas PVR, TIGIT, and complement pathways dominated in the *CASP1*^+^ state. In contrast, absolute information flow revealed stronger PDGF and VEGF signaling in *CASP1*^−^ cells, while SEMA3 and B7F exhibited enhanced activity in *CASP1*^+^ cells.

Finally, an integrated bubble-plot summary illustrated overall differences in intercellular signaling patterns between *CASP1*^+^ and *CASP1*^−^ populations, providing a comprehensive view of *CASP1*-dependent communication landscapes within the colonic immune microenvironment (Figure 6E).

### 3.9. Mendelian Randomization Analysis

**Figure 7:**
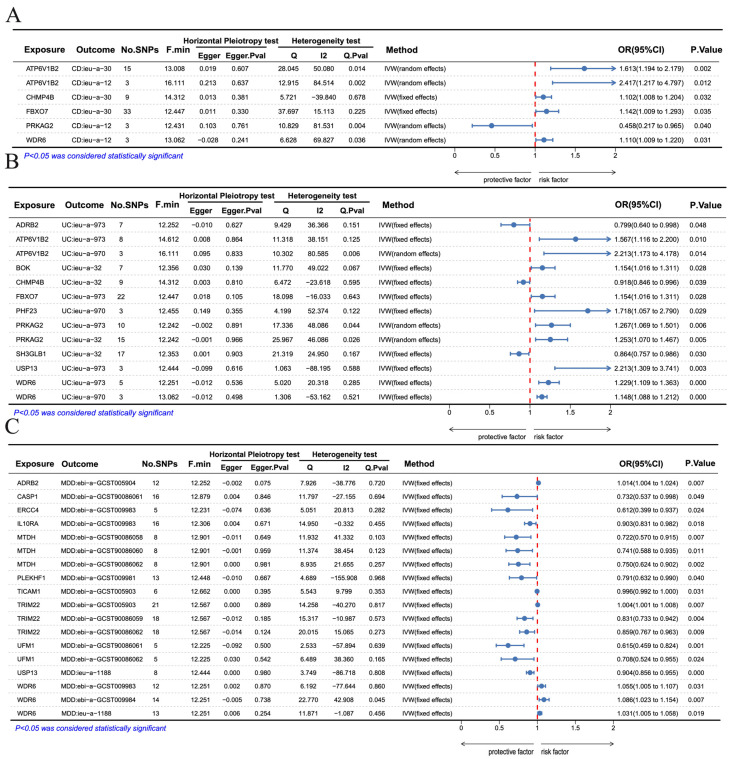
Mendelian randomization (MR) analysis evaluating the genetic causal effects of Co-DEGs associated with (**A**) CD, (**B**) UC, and (**C**) MDD.

To explore the genetic causal mechanisms underlying these diseases, we conducted Mendelian randomization (MR) analyses for CD (Figure 7A), UC (Figure 7B), and MDD (Figure 7C) using the IVW and MR-Egger methods. For CD, *ATP6V1B2*, *CHMP4B*, *FBXO7*, and *WDR6* were identified as causal risk factors, whereas *PRKAG2* exhibited a protective effect (Figure 7A). For UC, *ATP6V1B2*, *BOK*, *FBXO7*, *PHF23*, *PRKAG2*, *USP13,* and *WDR6* acted as risk factors, while *ADRB2*, *CHMP4B*, and *SH3GLB1* served as protective factors (Figure 7B). For MDD, *ADRB2*, and *WDR6* were identified as risk factors, whereas *CASP1*, *ERCC4*, *IL10RA*, *MTDH*, *PLEKHF1*, *TICAM1*, *TRIM22*, *UFM1*, and *USP13* demonstrated protective effects (Figure 7C). And leave-one-out analysis confirmed the robustness of the association (Supplementary Figures S4–S6). Overall, our analyses consistently reveal a set of autophagy-related genes exerting shared and disease-specific causal effects across CD, UC, and MDD, highlighting their central regulatory roles and potential as therapeutic targets.

## 4. Discussion

IBD is a chronic inflammatory disorder driven by complex interactions among host genetics, the gut microbiota, and mucosal immune responses [31]. Dysbiosis and impaired barrier integrity contribute to persistent intestinal inflammation, while autophagy plays a crucial role in maintaining epithelial homeostasis and regulating innate immune defense [32]. Disrupted autophagy has been repeatedly implicated in IBD pathogenesis, yet whether specific ARG alterations are causal or secondary to inflammation remains unclear [33].

MDD is likewise associated with chronic inflammation, neuroimmune dysregulation, and abnormal autophagy activity. Growing evidence suggests a bidirectional association between IBD and MDD, potentially mediated through immune activation and the gut–brain axis. However, the shared molecular mechanisms underlying this comorbidity have not been fully elucidated [34].

Given that ARGs influence both immune homeostasis and neuroinflammatory processes, this study sought to characterize their expression patterns across IBD and MDD and identify genes exhibiting consistent dysregulation. Initial profiling revealed 47 Co-DEGs shared across the two conditions, providing a basis for exploring their potential involvement in cross-disease biological pathways [35,36,37].

In this study, differential expression analyses were performed within a predefined set of ARGs rather than across the entire transcriptome. Because this gene set represents a biologically curated and functionally coherent group, applying genome-wide false discovery rate (FDR) correction would markedly reduce sensitivity and exclude biologically meaningful signals, particularly when integrating multiple tissues and datasets. Consistent with prior studies [38,39,40], we therefore reported nominal *p*-values to preserve analytical sensitivity within this restricted gene space. Nevertheless, when FDR correction was applied for validation, *CASP1* remained significantly dysregulated across UC and CD datasets, supporting the robustness of its identification (Supplementary Table S2). This approach balances statistical rigor with biological interpretability and is appropriate for mechanism-driven analyses centered on a targeted gene set rather than genome-wide discovery.

Comprehensive characterization of these 47 Co-DEGs revealed a compact protein–protein interaction network enriched for core autophagy regulators such as *BECN1*, *MAP1LC3B*, and *CASP1*. Further analysis revealed that these common genes displayed distinct co-expression characteristics between the disease groups and the control group, with the most significant changes observed in the prefrontal cortex and anterior cingulate cortex of MDD patients. By investigating the association between these genes and immune cell infiltration, we observed significant alterations in immune cell composition in the disease groups: the numbers of memory B cells, resting mast cells, and M1 macrophages were significantly reduced, while activated dendritic cells, neutrophils, and activated memory CD4^+^ T cells were significantly increased. These results suggest that the common genes may be involved in disease progression by regulating the distribution of immune cells.

Using machine learning methods, we screened *CASP1* as a SSAG for both IBD and MDD. *CASP1* serves as a pivotal regulatory factor in neurogenesis: it is indispensable to proper neurogenesis, and *CASP1* knock-out (*CASP1*-KO) induces cortical developmental defects arising from the impaired differentiation of radial glial cells [41]. While *CASP1* was identified as a key connecting biomarker, the underlying biological mechanisms linking these diseases must be interpreted with caution due to currently limited supporting evidence. The function of *CASP1* in neuropsychiatric or inflammatory pathways is not yet fully elucidated, with our results primarily depending on computational forecasting. These research advances advance our cross-field comprehension of the shared mechanisms between IBD and MDD. Our analyses revealed a strong co-expression pattern between *CASP1* and LYZ, suggesting that these two genes may participate in a coordinated regulatory network. Given their respective biological functions, this association is biologically plausible. LYZ encodes lysozyme, a key antimicrobial enzyme that contributes to innate immune defense by degrading bacterial cell walls [42]. When released from stressed or damaged cells, lysozyme can also function as a danger-associated molecular pattern (DAMP), thereby amplifying immune activation [43]. *CASP1*, in contrast, is a central effector of inflammasome activation and mediates pyroptosis, an inflammatory form of programmed cell death [44]. Pyroptotic cell lysis leads to the extracellular release of intracellular contents, including lysozyme [45]. Based on these interconnected roles, we infer that *CASP1* activation may indirectly regulate LYZ release through pyroptosis-associated mechanisms, creating a potential feed-forward inflammatory loop. While this proposed *CASP1*–LYZ regulatory link is consistent with their known biological functions, it remains a hypothesis derived from computational inference and should be validated experimentally.

Taken together, our MR analyses reveal both shared and disease-specific genetic causal patterns across CD, UC, and MDD. Several genes—including *ATP6V1B2*, *FBXO7*, *PHF23*, and *WDR6*—consistently emerged as risk factors in multiple conditions, suggesting that dysregulation of autophagy-related or immune-modulatory pathways may contribute broadly to the development of inflammatory and psychiatric disorders. In contrast, genes such as *PRKAG2*, *ADRB2*, *CHMP4B*, and *CASP1* demonstrated protective effects in specific disease contexts, indicating heterogeneous regulatory roles across tissues and phenotypes. These findings highlight a complex but biologically meaningful landscape in which autophagy-associated genes exert differential causal influences on IBD and MDD, providing mechanistic clues for understanding their comorbidity and identifying potential therapeutic targets. Our findings establish WDR6 as a cross-disorder risk gene consistently associated with Crohn’s disease, ulcerative colitis, and major depressive disorder. This repeated association indicates its involvement in fundamental biological mechanisms common to these conditions. The repeated association of *WDR6* suggests its potential involvement in biological mechanisms common to both conditions. As the *WDR6* protein contains WD40-repeat domains—structural motifs known to mediate protein-protein interactions within key cellular complexes [46]—it may influence fundamental processes such as autophagy regulation, cellular maintenance, and neural circuit function [47]. For instance, in inflammatory bowel disease, it is hypothesized that altered *WDR6* function could contribute to impaired autophagy, thereby affecting intestinal barrier integrity and immune homeostasis. Similarly, in major depressive disorder, it is possible that dysregulated *WDR6* activity might disturb neural plasticity and stress response pathways [48]. This shared mechanism provides a biological basis for the observed clinical comorbidity between these conditions. However, these mechanistic links remain speculative and require direct experimental validation. The proposed role of *WDR6* as a cross-disorder target is therefore preliminary, and any therapeutic implications for IBD-MDD comorbidity must be regarded as hypothetical until substantiated by further evidence. Further investigation of its precise molecular functions could reveal novel therapeutic strategies for managing IBD-MDD comorbidity.

## 5. Conclusions

In summary, by integrating bulk transcriptomics, single-cell profiling, drug–gene interaction analysis, and Mendelian randomization, our study provides a multilayered view of the shared molecular architecture linking IBD and MDD. At the RNA level, we identified *CASP1* as a SSAG with consistent dysregulation across tissues and strong functional involvement in immune remodeling. Single-cell analyses further demonstrated that *CASP1* reshapes the colonic immune microenvironment and modulates intercellular communication, while drug-response prediction and indicate that *CASP1* represents a potential candidate for therapeutic targeting.

At the genomic level, our Mendelian randomization findings revealed WDR6 as a reproducible genetic risk factor for CD, UC, and MDD, highlighting its potential role in shared susceptibility pathways underlying gut–brain axis disorders.

Together, these results suggest that *CASP1* represents an RNA-level actionable target, whereas *WDR6* reflects germline genetic liability, offering complementary therapeutic and mechanistic insights into IBD/MDD comorbidity. Future experimental studies and translational research will be essential to validate these findings and to develop targeted interventions based on these molecular signatures.

## 6. Limitations

Several limitations of this study should be noted. First, the MDD cohorts included here had relatively small sample sizes, which may limit the generalizability and robustness of our findings. Although these datasets capture key disease-relevant features, validation in larger and more diverse populations will be essential to strengthen the reliability of the conclusions. Second, the 47 Co-DEGs identified across IBD and MDD were obtained primarily from computational analyses using a nominal threshold of *p* < 0.05, which increases the risk of false positives. Accordingly, our DEG and Co-DEG findings should be viewed as exploratory and will require independent replication and experimental validation. Their biological functions and mechanistic roles remain to be confirmed experimentally. In future work, we plan to establish animal models of colitis, depression, and their comorbidity, and to examine tissue-specific expression changes of key targets such as *CASP1* using qPCR and Western blot analyses. Third, although our machine-learning models were internally validated using a training/test split, they were not assessed in an independent external cohort. Consequently, the cross-disease predictive value of *CASP1* should be interpreted with caution and requires further validation using external datasets. Fourth, the drug–gene correlation analysis was performed using the NCI-60 cancer cell line panel, which does not recapitulate the specific pathophysiology of IBD or MDD. Therefore, these associations should be considered exploratory and will require confirmation in disease-relevant biological systems. In subsequent studies, we aim to perform systematic cellular functional assays to evaluate the therapeutic potential of candidate compounds and to delineate their underlying molecular pathways in appropriate experimental models.

## Figures and Tables

**Table 1:** Differential immune cell infiltration in CD and UC.

	CD			UC		
Cell Type	Normal Samples	Disease Samples	*p*	Normal Samples	Disease Samples	*p*
B.cells.naive	0.0495 ± 0.0524	0.0252 ± 0.0431	0.0850	0.0303 ± 0.0429	0.0220 ± 0.0293	0.4852
B.cells.memory	0.0874 ± 0.0963	0.0393 ± 0.0545	0.0604	0.0877 ± 0.0815	0.0533 ± 0.0466	0.1366
Plasma.cells	0.0009 ± 0.0037	0.0053 ± 0.0119	0.0036	0.0010 ± 0.0030	0.0059 ± 0.0168	0.0426
T.cells.CD8	0.0205 ± 0.0266	0.0203 ± 0.0300	0.9877	0.0613 ± 0.0506	0.0508 ± 0.0473	0.4777
T.cells.CD4.naive	0.0000 ± 0.0000	0.0005 ± 0.0028	0.1050	0.0000 ± 0.0000	0.0000 ± 0.0000	NA
T.cells.CD4.memory.resting	0.1289 ± 0.0545	0.0873 ± 0.0571	0.0085	0.1177 ± 0.0735	0.0926 ± 0.0560	0.2321
T.cells.CD4.memory.activated	0.0847 ± 0.0433	0.1374 ± 0.0787	<0.001	0.1082 ± 0.0683	0.1217 ± 0.0697	0.5055
T.cells.follicular.helper	0.0015 ± 0.0063	0.0019 ± 0.0077	0.8196	0.0021 ± 0.0081	0.0036 ± 0.0103	0.5556
T.cells.regulatory.Tregs.	0.0255 ± 0.0309	0.0255 ± 0.0305	0.9935	0.0403 ± 0.0308	0.0299 ± 0.0275	0.2485
T.cells.gamma.delta	0.0365 ± 0.0327	0.0162 ± 0.0253	0.0248	0.0263 ± 0.0225	0.0220 ± 0.0293	0.5412
NK.cells.resting	0.0056 ± 0.0134	0.0215 ± 0.0277	<0.0010	0.0022 ± 0.0087	0.0198 ± 0.0235	<0.0010
NK.cells.activated	0.0352 ± 0.0323	0.0297 ± 0.0334	0.5263	0.0321 ± 0.0223	0.0225 ± 0.0394	0.2227
Monocytes	0.0000 ± 0.0000	0.0046 ± 0.0101	<0.0010	0.0015 ± 0.0059	0.0023 ± 0.0072	0.6884
Macrophages.M0	0.1455 ± 0.0519	0.1420 ± 0.0754	0.8155	0.1212 ± 0.0384	0.1425 ± 0.0722	0.1275
Macrophages.M1	0.0996 ± 0.0401	0.1003 ± 0.0368	0.9527	0.1019 ± 0.0431	0.0883 ± 0.0345	0.2726
Macrophages.M2	0.0901 ± 0.0412	0.0634 ± 0.0440	0.0231	0.0835 ± 0.0364	0.0798 ± 0.0485	0.7498
Dendritic.cells.resting	0.0210 ± 0.0265	0.0210 ± 0.0313	0.9997	0.0208 ± 0.0139	0.0146 ± 0.0226	0.1910
Dendritic.cells.activated	0.0049 ± 0.0107	0.0058 ± 0.0125	0.7626	0.0075 ± 0.0216	0.0100 ± 0.0155	0.6853
Mast.cells.resting	0.0813 ± 0.0527	0.1056 ± 0.1105	0.1590	0.0665 ± 0.0575	0.0426 ± 0.0580	0.1658
Mast.cells.activated	0.0385 ± 0.0342	0.0604 ± 0.0771	0.0600	0.0366 ± 0.0344	0.0965 ± 0.0799	<0.0010
Eosinophils	0.0000 ± 0.0000	0.0028 ± 0.0136	0.0426	0.0019 ± 0.0063	0.0050 ± 0.0131	0.1970
Neutrophils	0.0430 ± 0.0242	0.0839 ± 0.0541	<0.0010	0.0493 ± 0.0269	0.0744 ± 0.0474	0.0103

**Table 2:** Differential immune cell infiltration in MDD-blood, MDD-prefrontal cortex, MDD-anterior cingulate cortex, MDD-anterior amygdala.

	Blood			Prefrontal Cortex			Anterior Cingulate Cortex			Anterior Amygdala		
Cell Type	Normal Samples	Disease Samples	*p*	Normal Samples	Disease Samples	*p*	Normal Samples	Disease Samples	*p*	Normal Samples	Disease Samples	*p*
B.cells.naive	0.0153 ± 0.0159	0.0168 ± 0.0173	0.4336	0.0172 ± 0.0349	0.0218 ± 0.0323	0.7110	0.0184 ± 0.0324	0.0218 ± 0.0336	0.7930	0.0096 ± 0.0229	0.0117 ± 0.0238	0.7714
B.cells.memory	0.0181 ± 0.0168	0.0153 ± 0.0167	0.1426	0.0784 ± 0.0641	0.0679 ± 0.0838	0.7030	0.1112 ± 0.0958	0.1014 ± 0.1043	0.8060	0.0470 ± 0.0553	0.0539 ± 0.0750	0.7377
Plasma.cells	0.0008 ± 0.0024	0.0010 ± 0.0032	0.6447	0.0445 ± 0.0274	0.0452 ± 0.0188	0.9350	0.0454 ± 0.0324	0.0493 ± 0.0331	0.7660	0.1305 ± 0.0900	0.0819 ± 0.0789	0.0704
T.cells.CD8	0.2383 ± 0.0760	0.2368 ± 0.0917	0.8716	0.2689 ± 0.0542	0.2401 ± 0.1016	0.3440	0.2857 ± 0.0598	0.2790 ± 0.0836	0.8150	0.0953 ± 0.0717	0.0737 ± 0.0906	0.3972
T.cells.CD4.naive	0.0386 ± 0.0397	0.0377 ± 0.0354	0.8333	0.0541 ± 0.0515	0.0712 ± 0.0503	0.3640	0.0499 ± 0.0426	0.0439 ± 0.0314	0.6880	0.0096 ± 0.0283	0.0033 ± 0.0144	0.3653
T.cells.CD4.memory.resting	0.0120 ± 0.0316	0.0087 ± 0.0269	0.3254	0.0005 ± 0.0018	0.0130 ± 0.0446	0.2940	0.0043 ± 0.0093	0.0152 ± 0.0353	0.3020	0.1900 ± 0.1107	0.2308 ± 0.1135	0.2448
T.cells.CD4.memory.activated	0.0445 ± 0.0379	0.0468 ± 0.0377	0.5918	0.0000 ± 0.0000	0.0000 ± 0.0000	0.0000	0.0000 ± 0.0000	0.0000 ± 0.0000	0.0000	0.0000 ± 0.0000	0.0014 ± 0.0045	0.1791
T.cells.follicular.helper	0.0000 ± 0.0000	0.0000 ± 0.0002	0.3003	0.0414 ± 0.0505	0.0359 ± 0.0335	0.7300	0.0337 ± 0.0374	0.0505 ± 0.0438	0.3050	0.0494 ± 0.0432	0.0540 ± 0.0557	0.7670
T.cells.regulatory..Tregs.	0.0088 ± 0.0135	0.0085 ± 0.0137	0.8165	0.1014 ± 0.0504	0.0881 ± 0.0326	0.3980	0.0929 ± 0.0441	0.0992 ± 0.0498	0.7360	0.0233 ± 0.0470	0.0145 ± 0.0261	0.4562
T.cells.gamma.delta	0.0003 ± 0.0025	0.0005± 0.0050	0.5833	0.0000 ± 0.0000	0.0000 ± 0.0000	0.0000	0.0000 ± 0.0000	0.0000 ± 0.0000	0.0000	0.0023 ± 0.0077	0.0019 ± 0.0087	0.8670
NK.cells.resting	0.0670 ± 0.0315	0.0706 ± 0.031	0.3097	0.0353 ± 0.0461	0.0481 ± 0.0572	0.5050	0.0374 ± 0.0392	0.0316 ± 0.0434	0.7250	0.0378 ± 0.0457	0.0420 ± 0.0435	0.7630
NK.cells.activated	0.0290 ± 0.0314	0.0233 ± 0.0271	0.0934	0.0690 ± 0.0580	0.0722 ± 0.0802	0.9040	0.0497 ± 0.0596	0.0584 ± 0.0805	0.7580	0.0346 ± 0.0487	0.0522 ± 0.0636	0.3226
Monocytes	0.1650 ± 0.0590	0.1575 ± 0.0588	0.2598	0.0193 ± 0.0234	0.0206 ± 0.0274	0.8950	0.0182 ± 0.0223	0.0179 ± 0.0186	0.9700	0.0285 ± 0.0354	0.0532 ± 0.0499	0.0724
Macrophages.M0	0.0222 ± 0.0211	0.0315 ± 0.0254	<0.0010	0.0265 ± 0.0354	0.0340 ± 0.0346	0.5590	0.0137 ± 0.0228	0.0350 ± 0.0394	0.1090	0.0131 ± 0.0277	0.0324 ± 0.0483	0.1208
Macrophages.M1	0.0041 ± 0.0075	0.0041 ± 0.0092	0.9813	0.0417 ± 0.0325	0.0391 ± 0.0328	0.8280	0.0421 ± 0.0286	0.0238 ± 0.0279	0.1100	0.0074 ± 0.0153	0.0090 ± 0.0185	0.7628
Macrophages.M2	0.0066 ± 0.0098	0.0070 ± 0.0098	0.7026	0.1208 ± 0.0441	0.1072 ± 0.0374	0.3710	0.1247 ± 0.0360	0.1052 ± 0.0368	0.1850	0.2059 ± 0.0974	0.1466 ± 0.1161	0.0807
Dendritic.cells.resting	0.0001± 0.0006	0.0000 ± 0.0002	0.0950	0.0288 ± 0.0241	0.0202 ± 0.0204	0.3060	0.0186 ± 0.0172	0.0108 ± 0.0192	0.2860	0.0000 ± 0.0000	0.0005 ± 0.0023	0.3293
Dendritic.cells.activated	0.0033 ± 0.0072	0.0027 ± 0.0058	0.4366	0.0002 ± 0.0008	0.0001 ± 0.0003	0.5230	0.0000 ± 0.0000	0.0000 ± 0.0000	0.0000	0.0120 ± 0.0215	0.0095 ± 0.0154	0.6694
Mast.cells.resting	0.0115 ± 0.0138	0.0093 ± 0.0128	0.1598	0.0277 ± 0.0298	0.0435 ± 0.0379	0.2150	0.0224 ± 0.0321	0.0369 ± 0.0426	0.3400	0.0667 ± 0.0822	0.0980 ± 0.0871	0.2377
Mast.cells.activated	0.0354 ± 0.0549	0.0247 ± 0.0436	0.0641	0.0011 ± 0.0029	0.0025 ± 0.0096	0.6050	0.0063 ± 0.0180	0.0006 ± 0.0022	0.2780	0.0091 ± 0.0269	0.0120 ± 0.0336	0.7558
Eosinophils	0.0077 ± 0.0130	0.0104 ± 0.0152	0.0945	0.0110 ± 0.0217	0.0105 ± 0.0202	0.9410	0.0090 ± 0.0145	0.0033 ± 0.0082	0.2270	0.0124 ± 0.0213	0.0075 ± 0.0144	0.3856
Neutrophils	0.2714 ± 0.0970	0.2868 ± 0.1079	0.1806	0.0124 ± 0.0109	0.0190 ± 0.0287	0.4160	0.0162 ± 0.0126	0.0164 ± 0.0192	0.9790	0.0154 ± 0.0174	0.0100 ± 0.0215	0.3799

## Data Availability

The mRNA expression profiles were obtained from the GEO database (https://www.ncbi.nlm.nih.gov/geo/, accessed on 1 July 2025). The original contributions presented in this study are included in the article/Supplementary Materials. Further inquiries can be directed to the corresponding author(s).

## Supplementary Materials

The following supporting information can be downloaded at: https://www.mdpi.com/article/10.3390/genes17010004/s1. Figure S1. PCA plots show the fixation before batch processing in the UC (a), CD (c), and MDD (e) datasets, and the fixation after batch processing in the UC (b), CD (d), and MDD (f) datasets. Figure S2. Correlation matrices of the 47 Co-DEGs in control and disease groups across six sample types: (A) CD, (B) UC, (C) MDD whole blood, (D) MDD prefrontal cortex, (E) MDD anterior cingulate cortex, and (F) MDD amygdala. Figure S3. Correlations between the 47 Co-DEGs and immune cell infiltration across six sample types: (A) CD colon, (B) UC colon, (C) MDD whole blood, (D) MDD prefrontal cortex, (E) MDD anterior cingulate cortex, and (F) MDD amygdala. Figure S4. Leave-one-out sensitivity analysis of MR estimates for Co-DEGs associated with CD. (A, B) ATP6V1B2; (C) CHMP4B; (D) FBXO7; (E) PRKAG2; (F) WDR6. Figure S5. Leave-one-out sensitivity analysis of MR estimates for Co-DEGs associated with UC. (A, B) ADRB2; (C) ATP6V1B2; (D) BOK; (E) CHMP4B; (F) FBXO7; (G) PHF23; (H, I) PRKAG2; (J) SH3GLB1; (K) USP13; and (L, Z) WDR6. Figure S6. Leave-one-out sensitivity analysis of MR estimates for Co-DEGs associated with MDD. (A) ADRB2; (B) ATG7; (C) *CASP1*; (D) ERCC4; (E,F) IL10RA; (G–I) MTDH; (J) PLEKHF1; (K) TICAM1; (L–N) TRIM22; (O–Q) UFM1; (R–T) WDR6. Table S1. Autophagy-related genes are from the study Zou et al. Table S2. Nominal *p*-values and FDR-adjusted q-values for differential expression between normal and diseased samples in CD, UC, and MDD tissues (whole blood, prefrontal cortex, anterior cingulate cortex, and amygdala). Table S3. Accuracy, Precision, Recall, and F1-score of the models for Blood, CD, and UC samples.

## Abbreviations

The following abbreviations are used in this manuscript:

MDD	Major Depressive Disorder
IBD	Inflammatory Bowel Disease
DE-ARGs	Differentially Expressed Autophagy-related Genes
Co-DEGs	Co-Differentially Expressed Genes
CD	Crohn’s disease
UC	Ulcerative colitis
GEO	Gene Expression Omnibus
PPI	Protein-protein Interaction
SVM	Support Vector Machine
RF	Random Forest
GLM	Generalized Linear Model
XGB	eXtreme Gradient Boosting
GSVA	Gene Set Variation Analysis
PCA	Principal Component Analysis
GWAS	Genome-wide Association Studies
IVs	Instrumental variables
IVW	Inverse-variance weighted
SSAG	Cross-disease shared susceptibility-associated gene
