# Supplementary material for: Machine Learning Reveals Common Regulatory Mechanisms Mediated by Autophagy-Related Genes in the Development of Inflammatory Bowel Disease and Major Depressive Disorder
Source: Genes (Basel). 2025 Dec 19;17(1):4. doi: 10.3390/genes17010004 (PMC12841238; doi:10.3390/genes17010004)
Supplement: Supplementary file 1 [file genes-17-00004-s001.zip › genes-4032151-supplementary.pdf]

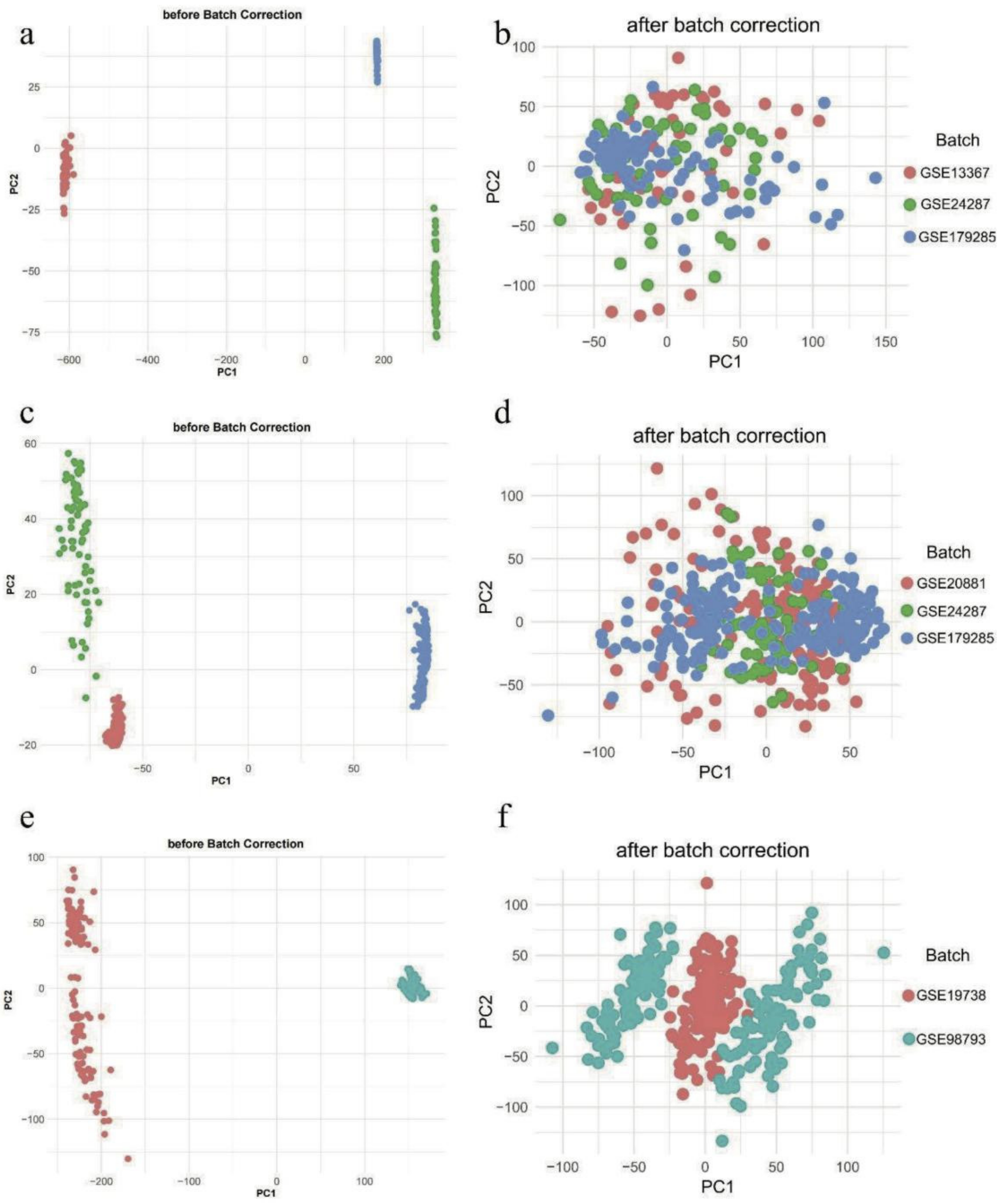

Figure S1. PCA plots show the fixation before batch processing in the UC (a), CD (c), and MDD (e) datasets, and the fixation after batch processing in the UC (b), CD (d), and MDD (f) datasets.

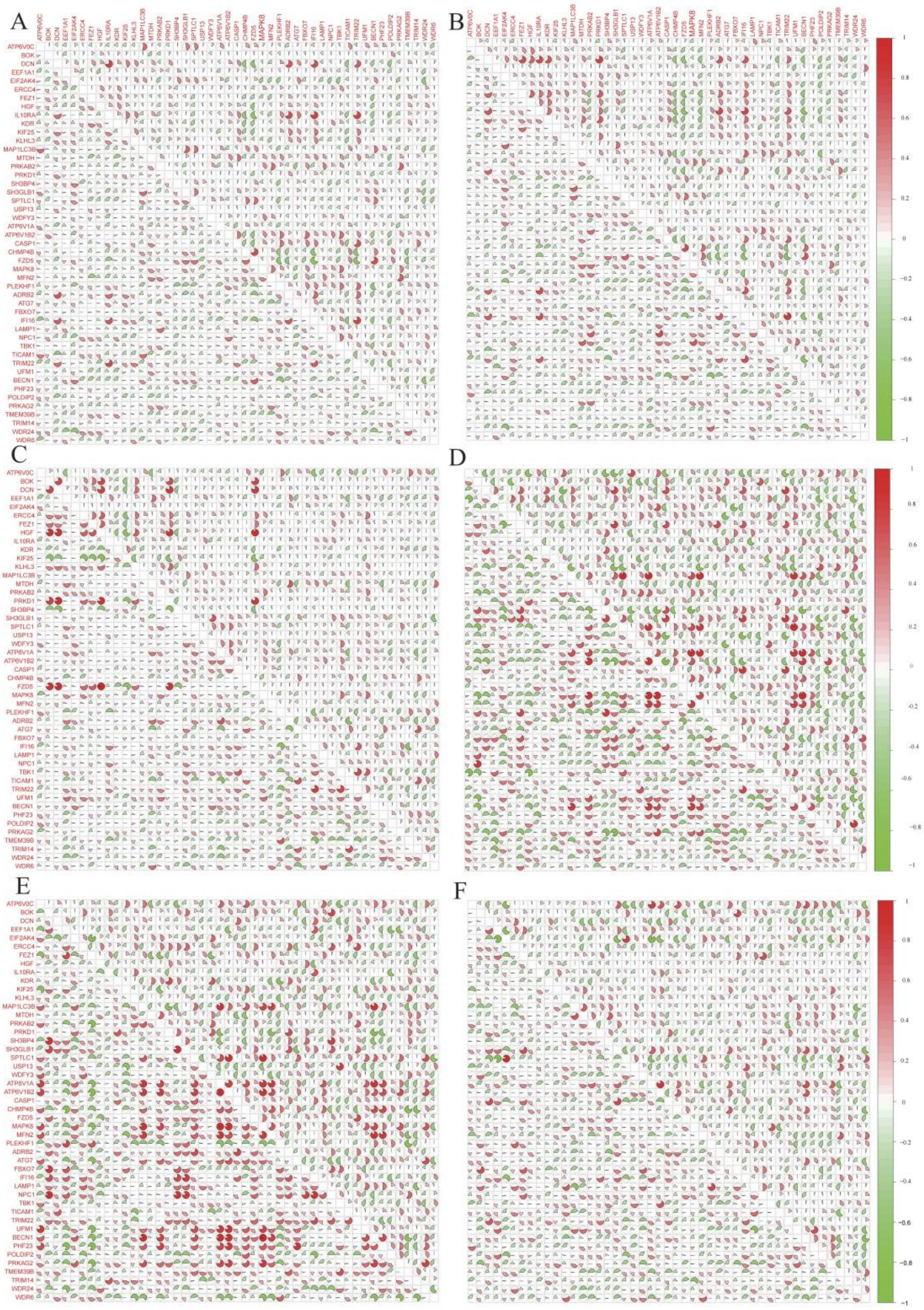

Figure S2. Correlation matrices of the 47 Co-DEGs in control and disease groups across six sample types: (A) CD, (B) UC, (C) MDD whole blood, (D) MDD prefrontal cortex, (E) MDD anterior cingulate cortex, and (F) MDD amygdala.

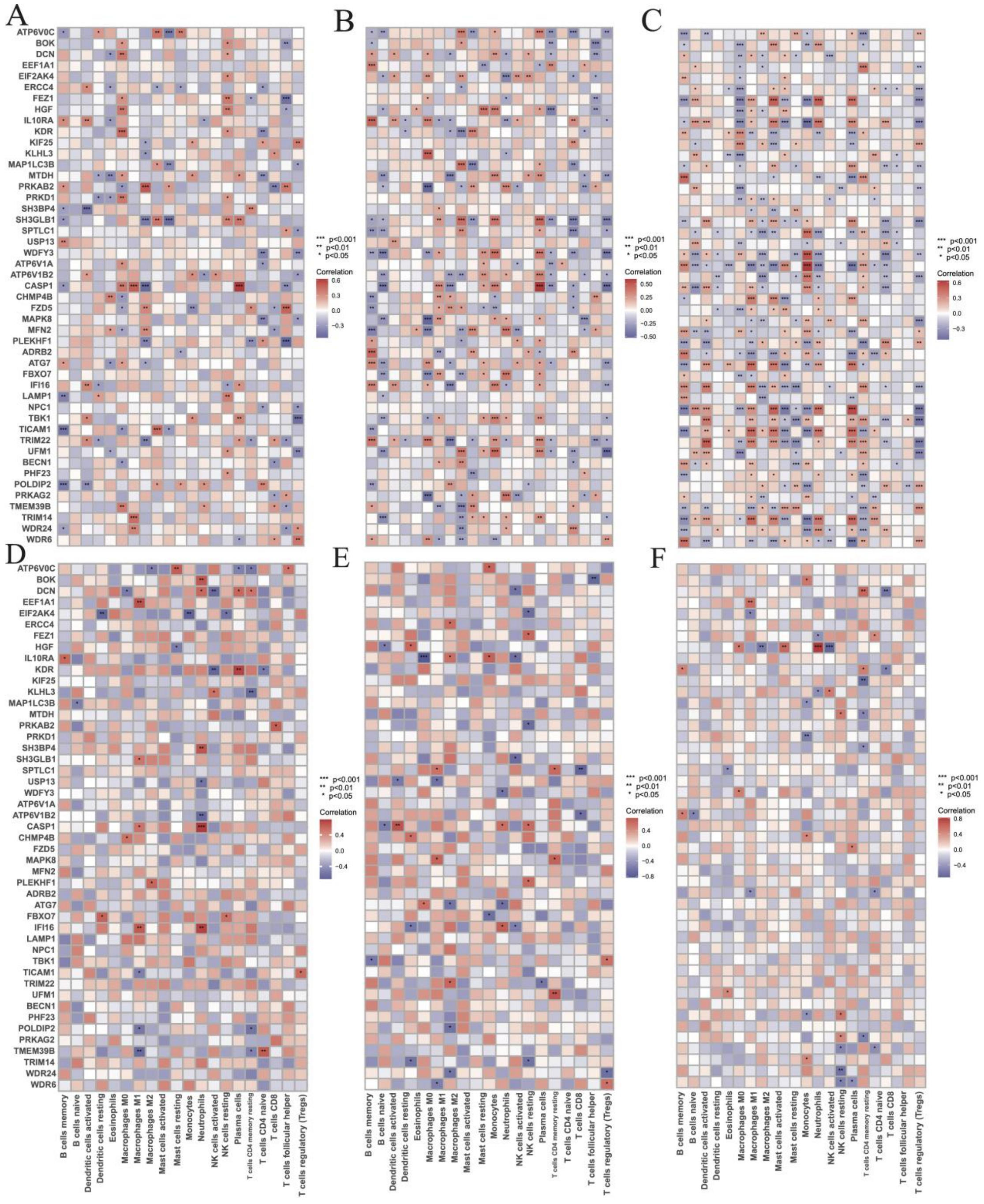

Figure S3. Correlations between the 47 Co-DEGs and immune cell infiltration across six sample types: (A) CD colon, (B) UC colon, (C) MDD whole blood, (D) MDD prefrontal cortex, (E) MDD anterior cingulate cortex, and (F) MDD amygdala.

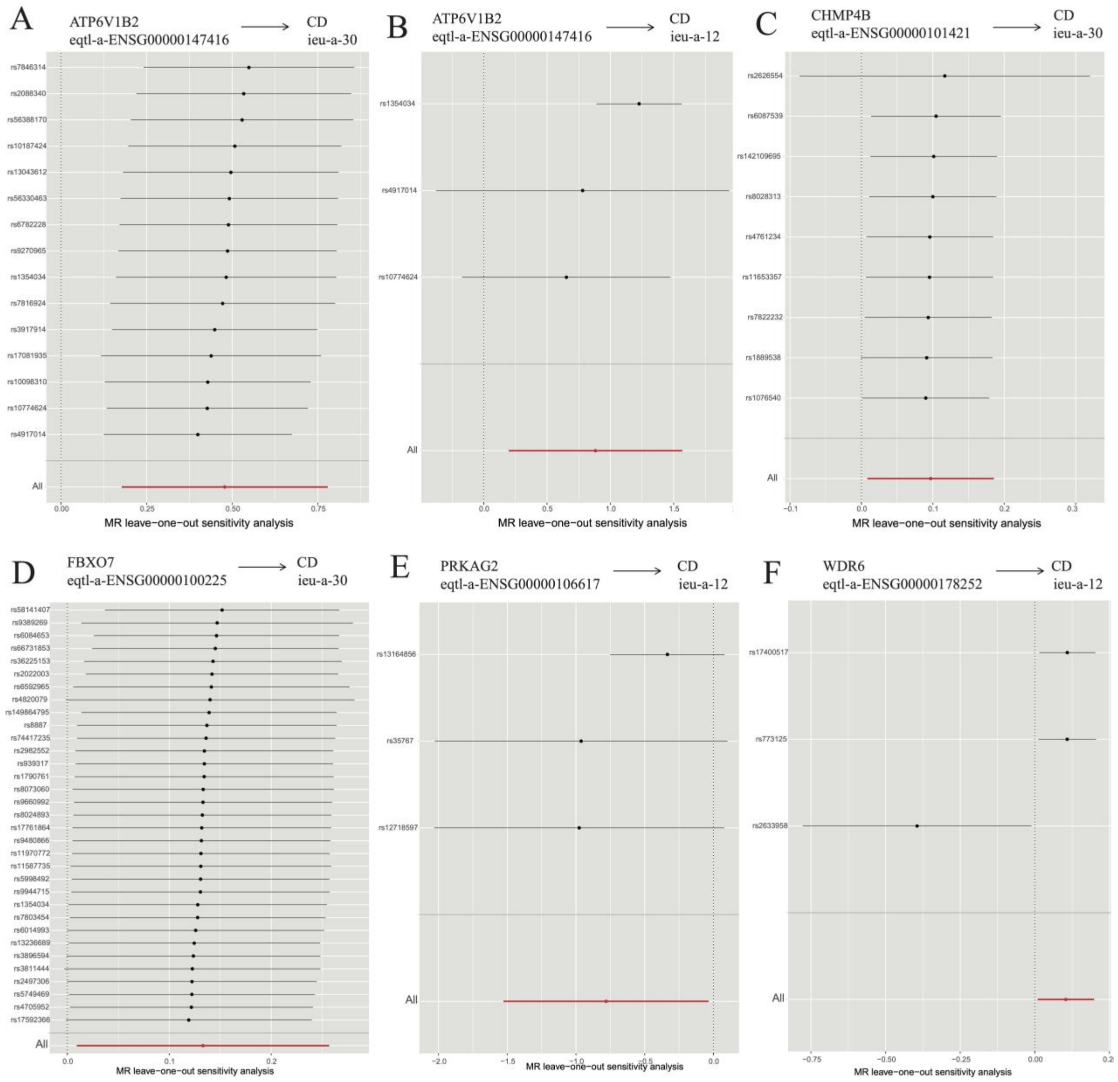

Figure S4. Leave-one-out sensitivity analysis of MR estimates for Co-DEGs associated with CD. (A, B) ATP6V1B2; (C) CHMP4B; (D) FBXO7; (E) PRKAG2; (F) WDR6.

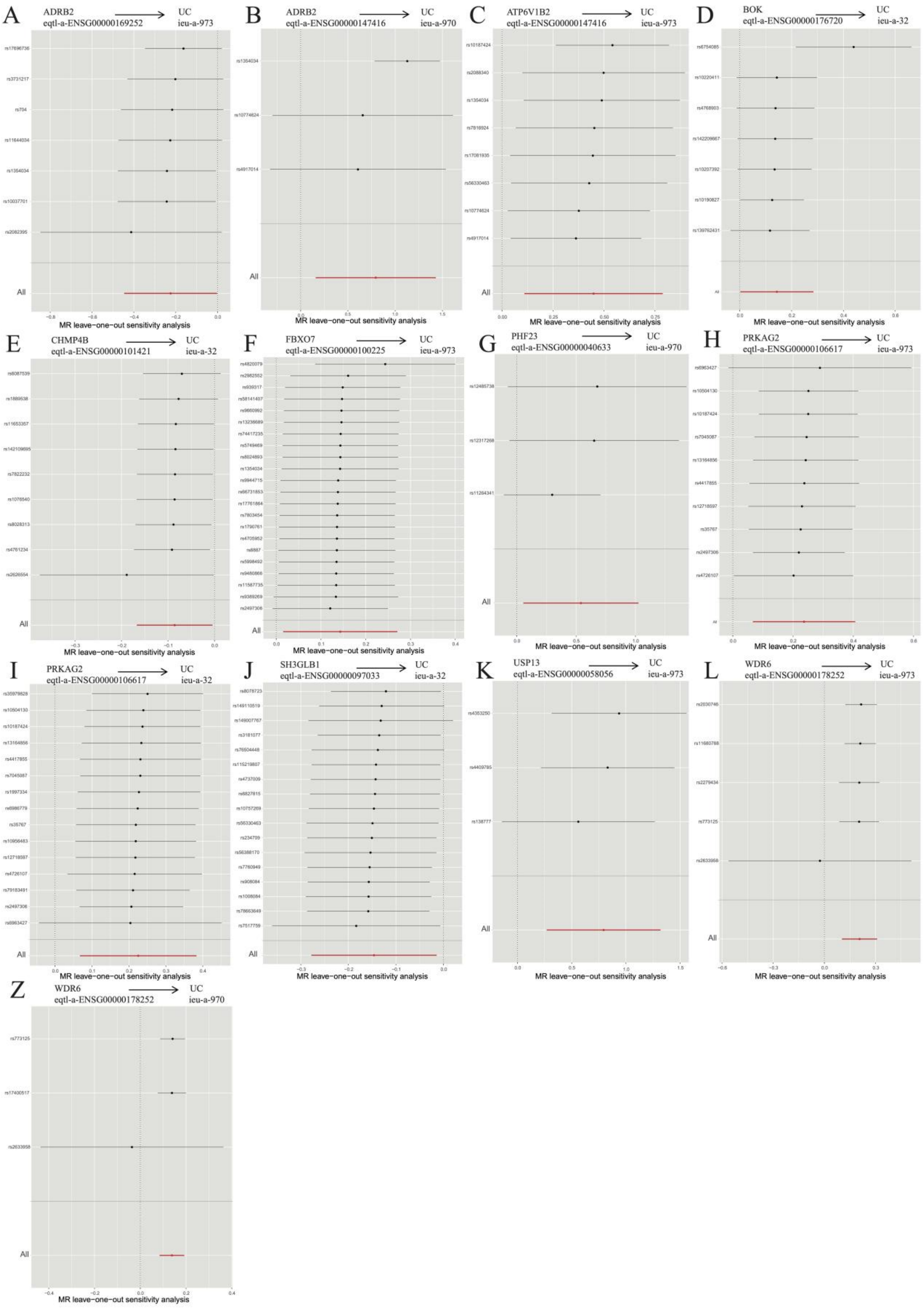

Figure S5. Leave-one-out sensitivity analysis of MR estimates for Co-DEGs associated with UC. (A, B) ADRB2; (C) ATP6V1B2; (D) BOK; (E) CHMP4B; (F) FBXO7; (G) PHF23; (H, I) PRKAG2; (J) SH3GLB1; (K) USP13; and (L, Z) WDR6.

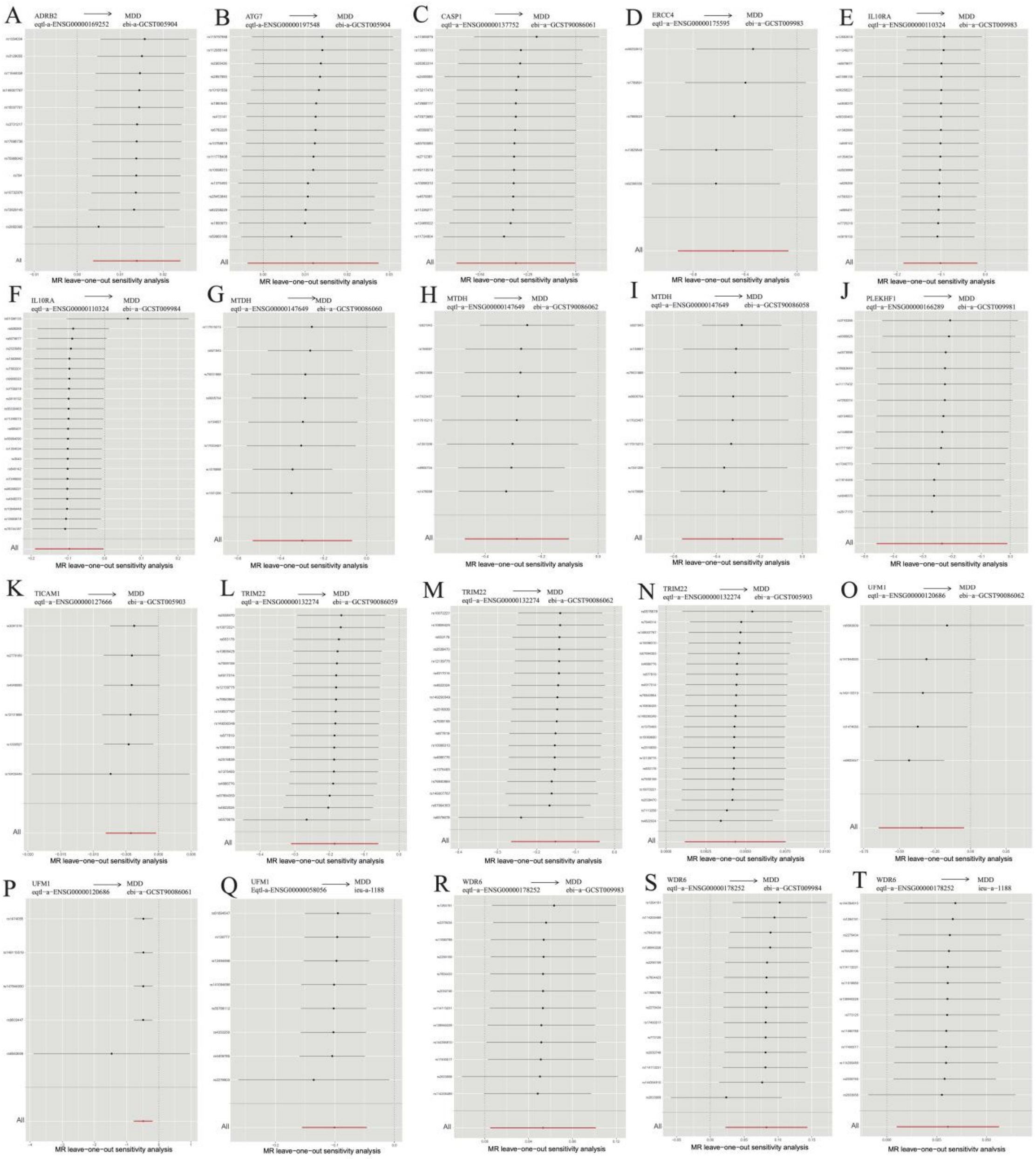

Figure S6. Leave-one-out sensitivity analysis of MR estimates for Co-DEGs associated with MDD. (A) ADRB2; (B) ATG7; (C) CASP1; (D) ERCC4; (E,F) IL10RA; (G–I) MTDH; (J) PLEKHF1; (K) TICAM1; (L–N) TRIM22; (O–Q) UFM1; (R–T) WDR6.

Table S1. Autophagy-related genes are from the study Zou et al.

ABL1	CAMKK2	FBXW7	KDM4A	NCOA4	RAB7A	STING1	UBQLN1
ABL2	CAPN1	FEZ1	KDR	NEDD4	RAB8A	STK11	UBQLN2
ACER2	CAPNS1	FEZ2	KEAP1	NLRP6	RALB	STUB1	UBQLN4
ADRA1A	CASP1	FLCN	KIF25	NOD1	RASIP1	SUPT5H	UCHL1
ADRB2	CASP3	FOXK1	KLHL22	NOD2	RB1CC1	SVIP	UFC1
AKT1	CDC37	FOXK2	KLHL3	NPC1	RETREG1	SYNPO2	UFL1
AMBRA1	CDK5	FOXO1	LACRT	NPRL2	RETREG3	TAB2	UFM1
ATF6	CDK5R1	FOXO3	LAMP1	NRBP2	RHEB	TAB3	ULK1
ATG101	CHMP4A	FTH1	LAMP2	NUPR1	RIPK2	TBC1D14	USP10
ATG13	CHMP4B	FTL	LAMP3	OPTN	RMC1	TBC1D25	USP13
ATG14	CISD2	FYCO1	LAMTOR1	ORMDL3	RNF152	TBK1	USP30
ATG2A	CLEC16A	FZD5	LAMTOR2	OSBPL7	RNF41	TEX264	USP33
ATG2B	CLN3	GAPDH	LAMTOR3	PAFAH1B2	RNF5	TFEB	USP36
ATG5	CLU	GATA4	LAMTOR4	PARK7	ROCK1	TICAM1	UVRAG
ATG7	CPTP	GBA	LAMTOR5	PHB2	RPTOR	TIGAR	VDAC1
ATM	CSNK2A2	GFAP	LARP1	PHF23	RRAGA	TLK2	VPS13C
ATP13A2	CTSA	GNAI3	LEP	PIK3C2A	RRAGB	TMEM150A	VPS13D
ATP6V0A1	CTTN	GOLGA2	LEPR	PIK3C3	RRAGC	TMEM150B	VPS26A
ATP6V0A2	DAP	GPR137	LGALS8	PIK3CA	RRAGD	TMEM150C	VPS26B
ATP6V0B	DAPK1	GPR137B	LRRK2	PIK3CB	RUBCN	TMEM39A	VPS35
ATP6V0C	DAPK2	GPSM1	LRSAM1	PIK3R2	RUFY4	TMEM39B	WAC
ATP6V0D1	DAPK3	GSK3A	LZTS1	PIM2	SCFD1	TMEM59	WASHC1
ATP6V0D2	DAPL1	GSK3B	MAP1LC3A	PINK1	SCOC	TOMM7	WDFY3

ATP6V0E1	DCN	HAX1	MAP1LC3B	PIP4K2A	SEC22B	TP53	WDR24
ATP6V0E2	DDIT3	HDAC6	MAP1LC3C	PIP4K2B	SESN1	TP53INP1	WDR41
ATP6V1A	DDRGK1	HERC1	MAP3K7	PIP4K2C	SESN2	TP53INP2	WDR6
ATP6V1B1	DEPDC5	HGF	MAPK15	PJVK	SESN3	TPCN1	WDR81
ATP6V1B2	DEPP1	HIF1A	MAPK3	PLEKHF1	SH3BP4	TPCN2	WIPI2
ATP6V1C1	DHRSX	HMGB1	MAPK8	PLK2	SH3GLB1	TREM2	ZC3H12A
ATP6V1C2	DNM1L	HMOX1	MAPT	PLK3	SIRT1	TRIB3	ZKSCAN3
ATP6V1D	DRAM1	HSP90AA1	MCL1	POLDIP2	SIRT2	TRIM13	ZMPSTE24
ATP6V1E1	DRAM2	HSPA8	MEFV	PRKAA1	SLC38A9	TRIM14	KIAA1324
ATP6V1E2	EEF1A1	HSPB1	MET	PRKAA2	SMCR8	TRIM21	
ATP6V1G1	EEF1A2	HSPB8	MFN2	PRKAB1	SMG1	TRIM22	
ATP6V1G2	EIF2AK4	HTR2B	MFSD8	PRKAB2	SNCA	TRIM27	
ATP6V1H	EIF4G1	HTRA2	MID2	PRKACA	SNRNP70	TRIM34	
AUP1	EIF4G2	HTT	MIR199A1	PRKAG1	SNX32	TRIM38	
BAD	ELAPOR1	HUWE1	MIRLET7B	PRKAG2	SNX5	TRIM5	
BAG3	EP300	IFI16	MLST8	PRKAG3	SNX6	TRIM6	
BCL2	EPM2A	IFNG	MT3	PRKD1	SOGA1	TRIM65	
BCL2L11	ERCC4	IKBKG	MTCL1	PRKN	SOGA3	TRIM68	
BECN1	ERN1	IL10	MTDH	PSAP	SPTLC1	TRIM8	
BMF	EXOC1	IL10RA	MTM1	PTPN22	SPTLC2	TRIML1	
BNIP3	EXOC4	IL4	MTMR3	PYCARD	SQSTM1	TRIML2	
BNIP3L	EXOC7	IRGM	MTMR4	QSOX1	SREBF1	TSC1	
BOK	EXOC8	ITPR1	MTMR8	RAB39B	SREBF2	TSC2	
C9orf72	FBXL2	KAT5	MTMR9	RAB3GAP1	STAT3	TSPO	
CALCOCO2	FBXO7	KAT8	MTOR	RAB3GAP2	STBD1	UBA5	

Table S2. Nominal p-values and FDR-adjusted q-values for differential expression between normal and diseased samples in CD, UC, and MDD tissues (whole blood, prefrontal cortex, anterior cingulate cortex, and amygdala).

	CD		UC		MDD-blood		MDD-prefrontal cortex		MDD-anterior cingulate cortex		MDD-anterior amygdala	
Gene	pvalue	FDR	pvalue	FDR	pvalue	FDR	pvalue	FDR	pvalue	FDR	pvalue	FDR
ABL1	0.0617	0.1380	0.1027	0.1988	0.8607	0.9485	0.5475	0.8715	0.1389	0.9077	0.3058	0.8714
ABL2	0.5388	0.6544	0.2129	0.3398	0.5924	0.8860	0.1300	0.6337	0.1389	0.9077	0.7652	0.9709
ACER2	NA	NA	NA	NA	NA	NA	NA	NA	NA	NA	NA	NA
ADRA1A	NA	NA	NA	NA	0.0647	0.2777	0.9025	0.9941	0.8798	0.9880	0.8110	0.9709
ADRB2	0.0028	0.0132	0.0402	0.1041	0.5456	0.8740	0.3892	0.7992	0.0441	0.7154	0.6361	0.9559
AKT1	0.3218	0.4506	0.0983	0.1953	0.8975	0.9667	0.3453	0.7584	0.6139	0.9719	0.3178	0.8714
AMBRA1	NA	NA	0.1589	0.2734	0.3543	0.7419	0.0186	0.6009	0.6498	0.9719	0.1516	0.8714
ATF6	0.1635	0.2649	0.0215	0.0634	0.9368	0.9838	0.2901	0.7191	0.7241	0.9719	0.4248	0.8714
ATG101	NA	NA	NA	NA	NA	NA	0.6827	0.9217	0.8403	0.9817	NA	NA
ATG13	NA	NA	0.0778	0.1647	0.6724	0.8894	0.4186	0.7998	0.4266	0.9719	NA	NA
ATG14	NA	NA	0.0691	0.1520	0.0655	0.2777	0.0145	0.6009	0.3897	0.9719	NA	NA
ATG2A	NA	NA	0.2716	0.4019	0.6156	0.8893	0.4067	0.7998	0.7196	0.9719	0.6721	0.9559
ATG2B	NA	NA	0.0560	0.1317	0.7915	0.9271	0.2454	0.7157	0.8010	0.9719	0.7436	0.9709
ATG5	0.0000	0.0003	0.0097	0.0362	0.3616	0.7432	0.1057	0.6337	0.8980	0.9942	0.6721	0.9559
ATG7	0.7643	0.8299	0.0071	0.0304	0.1706	0.5195	0.2671	0.7185	0.0387	0.7154	0.0048	0.8094
ATM	0.0702	0.1481	0.0193	0.0589	0.1868	0.5489	0.2328	0.7086	0.8798	0.9880	0.5333	0.9481
ATP13A2	0.0081	0.0284	0.0000	0.0002	0.8210	0.9355	0.2901	0.7191	0.2642	0.9077	0.1605	0.8714
ATP6V0A1	0.6565	0.7611	0.0056	0.0254	0.6535	0.8894	0.8195	0.9607	0.8575	0.9880	0.2905	0.8714

ATP6V0A2	0.8523	0.8925	0.9080	0.9291	0.6491	0.8894	0.9349	1.0000	0.3897	0.9719	0.8617	0.9709
ATP6V0B	0.0005	0.0038	0.0029	0.0149	0.7225	0.8894	0.2539	0.7163	1.0000	1.0000	0.7917	0.9709
ATP6V0C	0.4253	0.5595	0.0082	0.0325	0.0187	0.1389	1.0000	1.0000	0.6442	0.9719	0.0260	0.8094
ATP6V0D1	0.4457	0.5728	0.6555	0.7379	0.1338	0.4545	0.1147	0.6337	0.7195	0.9719	0.1605	0.8714
ATP6V0D2	0.1068	0.1932	0.2596	0.3947	0.0790	0.3113	0.0975	0.6337	0.4793	0.9719	0.6140	0.9559
ATP6V0E1	NA	NA	0.4571	0.5941	0.0148	0.1308	0.2017	0.6602	0.4793	0.9719	0.9405	0.9867
ATP6V0E2	NA	NA	0.5582	0.6608	0.2618	0.6448	0.5066	0.8388	0.5726	0.9719	0.0260	0.8094
ATP6V1A	0.0004	0.0037	0.2618	0.3947	0.4362	0.8048	0.0225	0.6009	0.6815	0.9719	0.1516	0.8714
ATP6V1B1	NA	NA	NA	NA	0.0050	0.0733	0.8063	0.9549	0.7196	0.9719	0.9298	0.9867
ATP6V1B2	0.0000	0.0003	0.4636	0.5970	0.0649	0.2777	0.0107	0.6009	0.4184	0.9719	0.3584	0.8714
ATP6V1C1	0.7365	0.8197	0.5599	0.6608	0.0601	0.2698	0.0814	0.6337	1.0000	1.0000	0.7842	0.9709
ATP6V1C2	NA	NA	NA	NA	0.0023	0.0733	0.1736	0.6371	0.9197	0.9942	0.4811	0.9027
ATP6V1D	0.0133	0.0426	0.1033	0.1988	0.9981	0.9986	0.8063	0.9549	1.0000	1.0000	0.1626	0.8714
ATP6V1E1	0.0916	0.1792	0.7631	0.8133	0.9821	0.9959	0.0213	0.6009	0.9386	1.0000	0.0893	0.8714
ATP6V1E2	0.1337	0.2294	0.0000	0.0002	0.7803	0.9271	0.3245	0.7266	0.3622	0.9719	0.6904	0.9689
ATP6V1G1	0.0255	0.0714	0.0005	0.0046	0.0587	0.2698	0.7437	0.9350	0.1857	0.9077	0.2130	0.8714
ATP6V1G2	0.0067	0.0239	0.1569	0.2715	0.3270	0.7191	0.0647	0.6337	0.7383	0.9719	0.3786	0.8714
ATP6V1H	0.0715	0.1487	0.9333	0.9456	0.3661	0.7439	0.0238	0.6009	0.3107	0.9458	0.1445	0.8714
AUP1	0.9119	0.9401	0.1111	0.2073	0.0275	0.1844	0.6185	0.8905	0.8798	0.9880	0.7723	0.9709
BAD	0.0015	0.0088	0.2448	0.3807	0.3285	0.7191	0.0892	0.6337	0.9387	1.0000	0.9801	0.9957
BAG3	0.0009	0.0059	0.0000	0.0000	0.5500	0.8740	0.9674	1.0000	0.6866	0.9719	0.6184	0.9559
BCL2	0.0042	0.0172	0.8235	0.8550	0.7873	0.9271	0.8063	0.9549	0.8010	0.9719	0.4545	0.9027
BCL2L11	0.0984	0.1870	0.0423	0.1086	0.5553	0.8740	0.8063	0.9549	0.0568	0.7154	0.3454	0.8714
BECN1	0.0365	0.0890	0.5431	0.6509	0.2693	0.6490	0.1370	0.6337	0.5446	0.9719	0.0209	0.8094
BMF	0.1327	0.2292	0.0019	0.0108	0.2333	0.6204	0.7437	0.9350	0.7241	0.9719	0.7917	0.9709
BNIP3	0.0907	0.1786	0.7419	0.7973	0.7261	0.8894	0.0970	0.6337	0.9591	1.0000	0.2629	0.8714

BNIP3L	0.0322	0.0821	0.5093	0.6298	0.6817	0.8894	0.0814	0.6337	0.5048	0.9719	0.8813	0.9725
BOK	0.3693	0.5064	0.0116	0.0415	0.0353	0.2187	0.6827	0.9217	0.0568	0.7154	0.4545	0.9027
C9orf72	0.1209	0.2158	0.0860	0.1778	0.8196	0.9355	0.0975	0.6337	0.1690	0.9077	0.9405	0.9867
CALCOCO2	0.0016	0.0088	0.0082	0.0325	0.7125	0.8894	0.2017	0.6602	0.7241	0.9719	0.4104	0.8714
CAMKK2	0.8286	0.8747	0.7359	0.7953	0.0079	0.0940	0.4305	0.7998	0.1857	0.9077	0.4248	0.8714
CAPN1	0.0309	0.0821	0.6478	0.7357	0.7407	0.8958	0.0502	0.6009	0.2035	0.9077	0.1682	0.8714
CAPNS1	0.2191	0.3293	0.0101	0.0371	0.9301	0.9803	0.3951	0.7998	0.6442	0.9719	0.2501	0.8714
CASP1	0.0000	0.0002	0.0003	0.0028	0.4885	0.8265	0.0408	0.6009	0.4483	0.9719	0.7652	0.9709
CASP3	0.7288	0.8149	0.0082	0.0325	0.8252	0.9371	0.6529	0.9062	0.3107	0.9458	0.4852	0.9027
CDC37	0.3120	0.4391	0.0071	0.0304	0.4444	0.8048	0.4067	0.7998	0.7005	0.9719	0.2627	0.8714
CDK5	0.0001	0.0007	0.4974	0.6203	0.5135	0.8516	0.1584	0.6371	0.5726	0.9719	0.5010	0.9161
CDK5R1	NA	NA	NA	NA	0.0001	0.0284	0.9674	1.0000	0.7241	0.9719	0.1994	0.8714
CHMP4A	0.7609	0.8299	0.1950	0.3178	NA	NA	0.1300	0.6337	0.5214	0.9719	0.7341	0.9709
CHMP4B	0.0408	0.0952	0.0019	0.0108	0.4341	0.8048	0.0344	0.6009	0.8980	0.9942	0.4248	0.8714
CISD2	NA	NA	0.1096	0.2064	0.2275	0.6145	0.5393	0.8664	0.8010	0.9719	0.3963	0.8714
CLEC16A	NA	NA	0.0079	0.0325	0.2669	0.6480	0.6529	0.9062	0.6139	0.9719	0.8813	0.9725
CLN3	0.0311	0.0821	0.7081	0.7728	0.8674	0.9496	0.8063	0.9549	1.0000	1.0000	0.7462	0.9709
CLU	0.1946	0.2991	0.0142	0.0467	0.4158	0.7858	0.6041	0.8905	0.7623	0.9719	0.3178	0.8714
CPTP	NA	NA	NA	NA	NA	NA	0.6529	0.9062	0.0955	0.9025	NA	NA
CSNK2A2	0.1764	0.2760	0.2491	0.3836	0.7234	0.8894	0.1064	0.6337	0.2642	0.9077	0.3302	0.8714
CTSA	NA	NA	0.0177	0.0555	0.5737	0.8816	0.6236	0.8905	0.5114	0.9719	0.3178	0.8714
CTTN	0.0028	0.0132	0.0232	0.0667	0.2805	0.6663	0.2133	0.6841	0.9197	0.9942	0.0682	0.8714
DAP	0.0000	0.0001	0.0212	0.0634	0.4151	0.7858	0.4807	0.8273	0.5214	0.9719	0.3557	0.8714
DAPK1	0.1710	0.2723	0.4412	0.5779	0.7009	0.8894	0.8381	0.9662	0.6139	0.9719	0.3963	0.8714
DAPK2	0.1022	0.1915	0.0001	0.0008	0.2120	0.5999	0.7437	0.9350	0.5214	0.9719	0.1079	0.8714
DAPK3	0.0007	0.0057	0.6279	0.7216	0.4383	0.8048	0.1873	0.6371	0.7241	0.9719	0.6721	0.9559

DAPL1	NA	NA	NA	NA	0.0004	0.0442	0.1064	0.6337	0.2869	0.9458	0.5460	0.9481
DCN	0.0208	0.0623	0.0064	0.0280	0.0090	0.1014	0.4363	0.7998	0.2642	0.9077	0.9799	0.9957
DDIT3	0.4218	0.5581	0.3142	0.4501	0.8064	0.9348	0.0026	0.4688	0.8175	0.9815	0.8617	0.9709
DDRGK1	NA	NA	0.9376	0.9468	0.0486	0.2550	0.9025	0.9941	0.9197	0.9942	NA	NA
DEPDC5	0.0000	0.0001	0.0005	0.0046	0.6649	0.8894	0.4610	0.7998	0.3897	0.9719	0.2018	0.8714
DEPP1	NA	NA	NA	NA	NA	NA	NA	NA	NA	NA	NA	NA
DHRSX	0.7291	0.8149	0.0107	0.0387	0.9171	0.9803	0.7437	0.9350	0.9598	1.0000	0.3650	0.8714
DNM1L	0.0085	0.0295	0.8245	0.8550	0.2131	0.5999	0.3892	0.7992	0.7005	0.9719	0.1530	0.8714
DRAM1	NA	NA	0.0001	0.0008	0.2561	0.6356	0.1710	0.6371	0.7241	0.9719	NA	NA
DRAM2	NA	NA	0.2991	0.4334	0.7384	0.8958	0.5949	0.8905	0.7241	0.9719	NA	NA
EEF1A1	0.0246	0.0706	0.0474	0.1188	0.0033	0.0733	0.3611	0.7810	0.7975	0.9719	0.9498	0.9901
EEF1A2	NA	NA	NA	NA	0.0036	0.0733	0.0892	0.6337	0.8010	0.9719	0.2791	0.8714
EIF2AK4	0.7417	0.8220	0.0004	0.0038	0.0172	0.1389	0.2328	0.7086	0.7241	0.9719	0.4697	0.9027
EIF4G1	0.0522	0.1196	0.9207	0.9359	0.4627	0.8158	0.4428	0.7998	0.5114	0.9719	0.2109	0.8714
EIF4G2	0.0175	0.0528	0.5153	0.6298	0.9870	0.9959	1.0000	1.0000	0.9598	1.0000	0.3718	0.8714
ELAPOR1	NA	NA	NA	NA	NA	NA	NA	NA	NA	NA	NA	NA
EP300	0.4615	0.5902	0.6176	0.7124	0.4738	0.8224	0.4610	0.7998	0.7196	0.9719	0.4545	0.9027
EPM2A	0.1047	0.1929	0.0880	0.1806	0.0049	0.0733	0.2997	0.7266	1.0000	1.0000	0.6184	0.9559
ERCC4	0.0025	0.0129	0.2464	0.3813	0.0098	0.1014	0.5125	0.8388	0.6261	0.9719	0.5837	0.9559
ERN1	0.1738	0.2736	0.1478	0.2586	0.1537	0.4951	0.8381	0.9662	0.2226	0.9077	0.5837	0.9559
EXOC1	0.0160	0.0495	0.0978	0.1953	0.4175	0.7858	0.2169	0.6841	0.2226	0.9077	0.3924	0.8714
EXOC4	0.9362	0.9498	0.7793	0.8277	0.0163	0.1366	0.0453	0.6009	0.6866	0.9719	0.3178	0.8714
EXOC7	0.0000	0.0003	0.5510	0.6578	0.5630	0.8811	0.4124	0.7998	0.6498	0.9719	0.3428	0.8714
EXOC8	0.9287	0.9498	0.4058	0.5541	0.3346	0.7191	0.2371	0.7154	0.2428	0.9077	0.8602	0.9709
FBXL2	NA	NA	NA	NA	0.0097	0.1014	0.6186	0.8905	0.7623	0.9719	0.1322	0.8714
FBXO7	0.0000	0.0001	0.1311	0.2348	0.9677	0.9918	0.0975	0.6337	0.0387	0.7154	0.1024	0.8714

FBXW7	0.0014	0.0085	0.0013	0.0089	0.2388	0.6251	0.0649	0.6337	0.2035	0.9077	0.9801	0.9957
FEZ1	0.1042	0.1929	0.0000	0.0001	0.0201	0.1403	0.6934	0.9326	0.2930	0.9458	0.7820	0.9709
FEZ2	0.0314	0.0821	0.0000	0.0002	0.9465	0.9908	0.3453	0.7584	0.2428	0.9077	0.9298	0.9867
FLCN	0.1721	0.2726	0.0489	0.1200	0.3444	0.7301	0.1370	0.6337	0.6444	0.9719	0.5667	0.9559
FOXK1	0.0042	0.0172	0.0140	0.0465	0.7952	0.9282	0.5949	0.8905	0.3897	0.9719	0.5377	0.9481
FOXK2	0.5259	0.6506	0.0910	0.1843	0.9306	0.9803	0.3245	0.7266	0.6498	0.9719	0.7462	0.9709
FOXO1	NA	NA	0.1706	0.2876	0.0604	0.2698	0.5949	0.8905	0.4483	0.9719	0.3825	0.8714
FOXO3	NA	NA	0.0243	0.0692	0.4362	0.8048	0.8034	0.9549	0.1857	0.9077	0.0315	0.8094
FTH1	0.0687	0.1477	0.1232	0.2218	0.7572	0.9124	0.3394	0.7550	0.4725	0.9719	0.9499	0.9901
FTL	0.0286	0.0777	0.0045	0.0214	0.9561	0.9918	0.5472	0.8715	0.5551	0.9719	0.0942	0.8714
FYCO1	0.0322	0.0821	0.0001	0.0010	0.6084	0.8893	0.4124	0.7998	0.7623	0.9719	0.5333	0.9481
FZD5	0.0000	0.0006	0.0009	0.0062	0.4855	0.8265	0.0329	0.6009	0.3358	0.9719	0.7529	0.9709
GAPDH	0.0172	0.0526	0.6036	0.7016	0.8092	0.9348	0.0695	0.6337	0.9790	1.0000	0.7532	0.9709
GATA4	NA	NA	NA	NA	0.0019	0.0733	0.1353	0.6337	0.5788	0.9719	0.8106	0.9709
GBA	0.1545	0.2537	0.0610	0.1361	NA	NA	0.5949	0.8905	0.3622	0.9719	0.5499	0.9481
GFAP	0.4270	0.5595	0.3823	0.5324	0.0059	0.0791	0.9674	1.0000	0.0642	0.7678	0.2826	0.8714
GNAI3	0.7746	0.8376	0.0018	0.0108	0.3241	0.7190	0.5125	0.8388	0.5114	0.9719	0.8800	0.9725
GOLGA2	0.0003	0.0026	0.4693	0.5997	0.4813	0.8265	0.9025	0.9941	0.9197	0.9942	0.6184	0.9559
GPR137	0.9009	0.9351	0.4202	0.5596	0.6310	0.8894	0.9025	0.9941	0.9598	1.0000	0.7247	0.9709
GPR137B	0.0000	0.0002	0.0172	0.0547	0.3132	0.7140	0.6041	0.8905	0.2226	0.9077	0.3963	0.8714
GPSM1	0.2225	0.3295	0.5136	0.6298	0.2427	0.6303	0.2328	0.7086	0.3897	0.9719	0.6361	0.9559
GSK3A	0.0380	0.0910	0.0019	0.0108	0.0600	0.2698	0.4610	0.7998	0.4483	0.9719	0.8617	0.9709
GSK3B	0.0020	0.0106	0.1987	0.3219	0.4738	0.8224	0.1160	0.6337	0.2183	0.9077	0.6598	0.9559
HAX1	0.4138	0.5503	0.9175	0.9357	0.0120	0.1119	0.1607	0.6371	0.8403	0.9817	0.8617	0.9709
HDAC6	0.2525	0.3670	0.0091	0.0349	0.5999	0.8860	0.4124	0.7998	0.6498	0.9719	0.0972	0.8714
HERC1	0.0324	0.0821	0.1181	0.2191	0.0570	0.2698	0.5393	0.8664	0.6139	0.9719	0.3854	0.8714

HGF	0.4377	0.5652	0.0097	0.0362	0.0430	0.2442	0.3245	0.7266	0.2035	0.9077	0.7627	0.9709
HIF1A	0.0051	0.0201	0.0000	0.0000	0.2656	0.6480	0.9835	1.0000	0.3107	0.9458	0.9801	0.9957
HMGB1	0.2250	0.3306	0.5144	0.6298	0.0283	0.1856	0.8195	0.9607	0.0292	0.7154	0.8228	0.9709
HMOX1	0.1243	0.2190	0.0018	0.0108	0.6671	0.8894	0.8063	0.9549	0.4483	0.9719	0.6598	0.9559
HSP90AA1	0.1304	0.2267	0.1613	0.2757	0.0026	0.0733	0.0867	0.6337	0.6416	0.9719	0.5885	0.9559
HSPA8	0.5979	0.7069	0.1542	0.2684	NA	NA	0.0557	0.6337	0.7194	0.9719	0.1322	0.8714
HSPB1	0.0855	0.1724	0.0967	0.1946	0.2035	0.5876	0.8063	0.9549	0.9197	0.9942	0.8800	0.9725
HSPB8	NA	NA	NA	NA	0.0028	0.0733	0.4305	0.7998	0.6139	0.9719	0.6361	0.9559
HTR2B	NA	NA	NA	NA	0.0096	0.1014	0.0235	0.6009	0.2642	0.9077	1.0000	1.0000
HTRA2	0.4101	0.5481	0.0521	0.1243	0.5716	0.8816	0.1485	0.6371	0.6816	0.9719	0.8034	0.9709
HTT	NA	NA	0.4966	0.6203	0.5077	0.8504	0.0295	0.6009	0.2281	0.9077	NA	NA
HUWE1	0.0642	0.1424	0.3589	0.5047	0.2167	0.6049	0.0852	0.6337	0.1007	0.9025	0.8813	0.9725
IFI16	0.0028	0.0132	0.0004	0.0037	0.4597	0.8149	0.2017	0.6602	0.0029	0.4974	0.3058	0.8714
IFNG	NA	NA	NA	NA	0.0182	0.1389	0.9010	0.9941	0.8010	0.9719	0.7242	0.9709
IKBKG	0.0088	0.0301	0.4210	0.5596	0.1233	0.4304	0.6236	0.8905	0.5788	0.9719	0.0826	0.8714
IL10	0.0934	0.1800	0.1005	0.1969	0.3616	0.7432	0.6529	0.9062	0.1857	0.9077	0.8307	0.9709
IL10RA	0.0057	0.0216	0.0133	0.0449	0.0461	0.2550	0.0502	0.6009	0.9598	1.0000	0.1702	0.8714
IL4	NA	NA	NA	NA	0.1785	0.5338	0.3245	0.7266	0.4793	0.9719	0.9799	0.9957
IRGM	NA	NA	NA	NA	NA	NA	1.0000	1.0000	0.4887	0.9719	0.5538	0.9481
ITPR1	0.1651	0.2649	0.0516	0.1243	0.3135	0.7140	0.4610	0.7998	0.0908	0.9025	0.5499	0.9481
KAT5	NA	NA	0.2619	0.3947	0.5945	0.8860	0.1736	0.6371	0.7623	0.9719	NA	NA
KAT8	NA	NA	0.0161	0.0516	0.6457	0.8894	1.0000	1.0000	0.4184	0.9719	NA	NA
KDM4A	NA	NA	0.0013	0.0086	0.9846	0.9959	0.1485	0.6371	0.1254	0.9065	NA	NA
KDR	0.0010	0.0063	0.0817	0.1712	0.0041	0.0733	0.1736	0.6371	0.1129	0.9065	0.0275	0.8094
KEAP1	0.0009	0.0059	0.0772	0.1647	0.9114	0.9785	0.1160	0.6337	0.3107	0.9458	0.1024	0.8714
KIF25	0.0347	0.0854	0.2184	0.3467	0.0031	0.0733	0.1873	0.6371	0.8777	0.9880	0.8228	0.9709

KLHL22	0.5915	0.7025	0.2635	0.3947	0.2762	0.6608	0.2901	0.7191	0.8403	0.9817	0.2272	0.8714
KLHL3	0.1208	0.2158	0.0118	0.0417	0.0022	0.0733	0.8063	0.9549	0.0051	0.4974	0.2606	0.8714
LACRT	NA	NA	NA	NA	0.0049	0.0733	1.0000	1.0000	0.8798	0.9880	0.2620	0.8714
LAMP1	0.0265	0.0733	0.4766	0.6066	0.9262	0.9803	0.2671	0.7185	0.0295	0.7154	0.6416	0.9559
LAMP2	0.2276	0.3327	0.1087	0.2064	0.2331	0.6204	0.1607	0.6371	0.0086	0.4974	0.3825	0.8714
LAMP3	0.2770	0.3982	0.0250	0.0698	0.1628	0.5056	0.4363	0.7998	0.7241	0.9719	0.8999	0.9862
LAMTOR1	NA	NA	0.4652	0.5970	0.8650	0.9496	0.9835	1.0000	0.2642	0.9077	NA	NA
LAMTOR2	NA	NA	0.5274	0.6370	0.4881	0.8265	0.8195	0.9607	0.1118	0.9065	NA	NA
LAMTOR3	NA	NA	0.5761	0.6773	0.8177	0.9355	0.2627	0.7185	0.7583	0.9719	NA	NA
LAMTOR4	NA	NA	NA	NA	NA	NA	0.3245	0.7266	0.2226	0.9077	NA	NA
LAMTOR5	NA	NA	NA	NA	NA	NA	0.0852	0.6337	0.4570	0.9719	NA	NA
LARP1	0.0481	0.1112	0.6564	0.7379	0.5932	0.8860	0.7437	0.9350	0.4184	0.9719	0.0575	0.8714
LEP	NA	NA	NA	NA	0.0030	0.0733	0.2496	0.7157	0.3358	0.9719	0.4248	0.8714
LEPR	0.0707	0.1481	0.6440	0.7347	0.8740	0.9537	0.8381	0.9662	0.2226	0.9077	0.1844	0.8714
LGALS8	0.0224	0.0663	0.3837	0.5324	0.8318	0.9383	0.6236	0.8905	0.7241	0.9719	0.9207	0.9867
LRRK2	0.0053	0.0203	0.0053	0.0245	0.0785	0.3113	0.4864	0.8273	0.8010	0.9719	0.9405	0.9867
LRSAM1	0.0000	0.0003	0.3540	0.5002	0.4992	0.8403	0.2854	0.7191	0.2226	0.9077	0.2501	0.8714
LZTS1	0.5915	0.7025	0.1996	0.3219	0.0002	0.0414	1.0000	1.0000	0.2428	0.9077	0.3302	0.8714
MAP1LC3A	0.4329	0.5645	0.4157	0.5591	0.7813	0.9271	0.9835	1.0000	0.7196	0.9719	0.4504	0.9027
MAP1LC3B	0.0045	0.0182	0.0000	0.0001	0.0375	0.2283	0.0058	0.6009	0.8174	0.9815	0.8422	0.9709
MAP1LC3C	NA	NA	NA	NA	0.0045	0.0733	0.9349	1.0000	1.0000	1.0000	0.8013	0.9709
MAP3K7	0.3068	0.4347	0.4066	0.5541	0.5206	0.8536	0.1873	0.6371	0.7241	0.9719	0.2826	0.8714
MAPK15	NA	NA	NA	NA	0.7089	0.8894	0.4610	0.7998	0.4483	0.9719	0.0848	0.8714
MAPK3	0.6609	0.7611	0.0281	0.0765	0.1567	0.4999	0.7437	0.9350	0.4793	0.9719	0.3786	0.8714
MAPK8	0.0884	0.1755	0.0063	0.0280	0.6041	0.8876	0.0295	0.6009	0.1857	0.9077	0.6148	0.9559
MAPT	NA	NA	NA	NA	0.0025	0.0733	0.4864	0.8273	0.8980	0.9942	0.3963	0.8714

MCL1	0.0029	0.0132	0.0002	0.0020	0.2251	0.6145	0.4363	0.7998	0.3622	0.9719	0.1844	0.8714
MEFV	0.6091	0.7138	0.0905	0.1843	0.5316	0.8603	0.4186	0.7998	0.4184	0.9719	0.8404	0.9709
MET	0.1018	0.1915	0.0019	0.0108	0.0600	0.2698	0.7130	0.9350	0.9598	1.0000	0.8404	0.9709
MFN2	0.0001	0.0006	0.0000	0.0000	0.0908	0.3420	0.0164	0.6009	0.3050	0.9458	0.4852	0.9027
MFSD8	NA	NA	0.0044	0.0214	0.3833	0.7568	0.1465	0.6371	0.3694	0.9719	0.3058	0.8714
MID2	0.2013	0.3059	0.0718	0.1557	0.0112	0.1076	0.3245	0.7266	0.2035	0.9077	0.2826	0.8714
MIR199A1	NA	NA	NA	NA	NA	NA	NA	NA	NA	NA	NA	NA
MIRLET7B	NA	NA	NA	NA	NA	NA	NA	NA	NA	NA	NA	NA
MLST8	NA	NA	0.4428	0.5779	0.5991	0.8860	0.6783	0.9217	0.2428	0.9077	NA	NA
MT3	0.0000	0.0003	0.1222	0.2218	0.9282	0.9803	0.1353	0.6337	0.6815	0.9719	0.5602	0.9535
MTCL1	NA	NA	NA	NA	NA	NA	0.0502	0.6009	0.1254	0.9065	NA	NA
MTDH	0.0034	0.0144	0.0023	0.0123	0.0148	0.1308	0.1485	0.6371	0.8798	0.9880	0.9207	0.9867
MTM1	0.0036	0.0151	0.0398	0.1041	0.5729	0.8816	0.5393	0.8664	0.5788	0.9719	0.7088	0.9709
MTMR3	0.0008	0.0059	0.0012	0.0086	0.0601	0.2698	0.2854	0.7191	0.8798	0.9880	0.2501	0.8714
MTMR4	0.8983	0.9351	0.0324	0.0861	0.7275	0.8894	0.6783	0.9217	0.6498	0.9719	0.8111	0.9709
MTMR8	0.0000	0.0003	NA	NA	NA	NA	NA	NA	NA	NA	0.3514	0.8714
MTMR9	0.0015	0.0087	0.9693	0.9724	0.9986	0.9986	0.0779	0.6337	0.1534	0.9077	0.9207	0.9867
MTOR	NA	NA	0.0129	0.0441	0.0689	0.2884	0.6236	0.8905	0.5114	0.9719	NA	NA
NCOA4	0.2835	0.4054	0.0001	0.0008	0.3690	0.7446	0.4935	0.8313	0.1909	0.9077	0.3923	0.8714
NEDD4	0.0052	0.0203	0.6421	0.7347	0.0546	0.2698	0.1370	0.6337	1.0000	1.0000	0.4734	0.9027
NLRP6	NA	NA	0.1228	0.2218	0.3863	0.7568	1.0000	1.0000	0.1857	0.9077	0.1944	0.8714
NOD1	NA	NA	0.2647	0.3947	0.9923	0.9982	0.9349	1.0000	0.5902	0.9719	0.6237	0.9559
NOD2	NA	NA	0.0270	0.0741	0.1343	0.4545	0.5668	0.8905	0.8010	0.9719	1.0000	1.0000
NPC1	0.0317	0.0821	0.2727	0.4019	0.1422	0.4727	0.6529	0.9062	0.0387	0.7154	0.1844	0.8714
NPRL2	NA	NA	0.0016	0.0106	0.3813	0.7568	0.1198	0.6337	0.1857	0.9077	NA	NA
NRBP2	0.2179	0.3293	NA	NA	NA	NA	NA	NA	NA	NA	0.7462	0.9709

NUPR1	0.1293	0.2263	0.0209	0.0630	0.8879	0.9647	0.7437	0.9350	0.8798	0.9880	0.8228	0.9709
OPTN	0.5286	0.6509	0.0245	0.0692	0.9628	0.9918	1.0000	1.0000	0.3426	0.9719	0.5799	0.9559
ORMDL3	0.0135	0.0426	0.2997	0.4334	0.5249	0.8536	0.7748	0.9549	0.8403	0.9817	0.2629	0.8714
OSBPL7	NA	NA	NA	NA	0.0100	0.1014	0.3245	0.7266	0.8798	0.9880	0.4852	0.9027
PAFAH1B2	0.3072	0.4347	0.0491	0.1200	0.2862	0.6751	0.0892	0.6337	0.6498	0.9719	0.6325	0.9559
PARK7	0.6874	0.7815	0.0836	0.1740	0.0184	0.1389	0.3831	0.7992	0.6808	0.9719	0.3143	0.8714
PHB2	0.5887	0.7025	0.0008	0.0058	0.0823	0.3206	0.5069	0.8388	0.4266	0.9719	0.5499	0.9481
PHF23	0.0001	0.0007	0.0001	0.0010	0.2889	0.6768	0.1873	0.6371	0.3622	0.9719	0.0209	0.8094
PIK3C2A	0.0061	0.0226	0.0047	0.0222	0.0934	0.3475	0.9674	1.0000	0.5788	0.9719	0.8422	0.9709
PIK3C3	0.0006	0.0048	0.0002	0.0017	0.7175	0.8894	0.5949	0.8905	0.9197	0.9942	0.9298	0.9867
PIK3CA	0.0009	0.0059	0.4941	0.6203	0.7901	0.9271	0.1873	0.6371	0.1389	0.9077	0.6009	0.9559
PIK3CB	0.4870	0.6139	0.4141	0.5591	0.6505	0.8894	0.9025	0.9941	0.2035	0.9077	0.1844	0.8714
PIK3R2	NA	NA	NA	NA	0.1430	0.4727	0.1261	0.6337	0.2226	0.9077	0.3854	0.8714
PIM2	0.0000	0.0003	0.0025	0.0132	0.6418	0.8894	0.6236	0.8905	0.6139	0.9719	0.4136	0.8714
PINK1	0.0000	0.0001	0.0006	0.0049	0.3863	0.7568	0.9669	1.0000	0.5900	0.9719	0.0871	0.8714
PIP4K2A	NA	NA	0.0098	0.0362	0.3584	0.7432	0.4610	0.7998	0.0568	0.7154	NA	NA
PIP4K2B	NA	NA	0.0592	0.1343	0.0046	0.0733	0.6236	0.8905	0.5114	0.9719	0.7842	0.9709
PIP4K2C	NA	NA	0.0569	0.1328	0.3176	0.7140	0.1160	0.6337	0.5446	0.9719	0.3428	0.8714
PJVK	NA	NA	NA	NA	NA	NA	NA	NA	NA	NA	NA	NA
PLEKHF1	0.0000	0.0000	0.2513	0.3850	0.8517	0.9459	0.0128	0.6009	0.7241	0.9719	0.0358	0.8094
PLK2	0.0927	0.1800	0.0121	0.0418	0.2469	0.6313	0.8034	0.9549	0.2281	0.9077	0.6781	0.9601
PLK3	0.5383	0.6544	0.0017	0.0106	0.3886	0.7569	0.3046	0.7266	0.5553	0.9719	0.8011	0.9709
POLDIP2	0.0029	0.0132	0.0709	0.1550	0.6431	0.8894	0.9349	1.0000	0.4793	0.9719	0.0325	0.8094
PRKAA1	0.5510	0.6662	0.4628	0.5970	0.3471	0.7314	0.3669	0.7810	0.6444	0.9719	0.8422	0.9709
PRKAA2	NA	NA	NA	NA	0.0484	0.2550	0.3245	0.7266	0.1014	0.9025	0.6505	0.9559
PRKAB1	0.5369	0.6544	0.0581	0.1343	0.6706	0.8894	0.9025	0.9941	0.6866	0.9719	0.2501	0.8714

PRKAB2	0.9430	0.9502	0.0000	0.0007	0.0311	0.2002	0.6236	0.8905	0.8403	0.9817	0.6540	0.9559
PRKACA	0.6606	0.7611	0.7051	0.7728	0.0471	0.2550	0.9349	1.0000	0.1254	0.9065	0.9398	0.9867
PRKAG1	0.8097	0.8697	0.8039	0.8451	0.5209	0.8536	0.1370	0.6337	0.7241	0.9719	0.6009	0.9559
PRKAG2	0.3329	0.4636	0.0252	0.0698	0.3936	0.7623	0.5125	0.8388	0.7583	0.9719	0.0182	0.8094
PRKAG3	NA	NA	NA	NA	0.0008	0.0562	0.7437	0.9350	0.9795	1.0000	0.6685	0.9559
PRKD1	0.5121	0.6396	0.0216	0.0634	0.0178	0.1389	0.0408	0.6009	0.2226	0.9077	0.0994	0.8714
PRKN	NA	NA	NA	NA	NA	NA	NA	NA	NA	NA	NA	NA
PSAP	0.0000	0.0000	0.0433	0.1102	0.6671	0.8894	0.5893	0.8905	0.8777	0.9880	0.1548	0.8714
PTPN22	0.0015	0.0087	0.9809	0.9809	0.9638	0.9918	0.8034	0.9549	0.5788	0.9719	0.8013	0.9709
PYCARD	0.5222	0.6491	0.2942	0.4303	0.8898	0.9647	0.2901	0.7191	0.1254	0.9065	0.2109	0.8714
QSOX1	NA	NA	0.0000	0.0005	0.1290	0.4456	0.0742	0.6337	1.0000	1.0000	0.7088	0.9709
RAB39B	NA	NA	NA	NA	0.5903	0.8860	0.7130	0.9350	0.3897	0.9719	0.4580	0.9027
RAB3GAP1	0.1543	0.2537	0.0294	0.0795	0.1453	0.4727	0.9009	0.9941	0.0568	0.7154	0.6506	0.9559
RAB3GAP2	0.1892	0.2925	0.0019	0.0108	0.9628	0.9918	1.0000	1.0000	0.4184	0.9719	NA	NA
RAB7A	NA	NA	0.3999	0.5523	0.6400	0.8894	0.7437	0.9350	0.8010	0.9719	0.9699	0.9957
RAB8A	0.0825	0.1689	0.0781	0.1647	0.3340	0.7191	0.6236	0.8905	0.3107	0.9458	0.1079	0.8714
RALB	0.7059	0.7990	0.0056	0.0254	0.7166	0.8894	0.2539	0.7163	0.2642	0.9077	0.3854	0.8714
RASIP1	0.0000	0.0001	0.0006	0.0050	0.3824	0.7568	0.3892	0.7992	0.8010	0.9719	0.4852	0.9027
RB1CC1	0.1051	0.1929	0.0089	0.0346	0.6215	0.8894	0.5949	0.8905	0.8374	0.9817	0.6540	0.9559
RETREG1	NA	NA	NA	NA	NA	NA	NA	NA	NA	NA	NA	NA
RETREG3	NA	NA	NA	NA	NA	NA	NA	NA	NA	NA	NA	NA
RHEB	0.1062	0.1932	0.4874	0.6152	0.7307	0.8901	0.2169	0.6841	0.5788	0.9719	0.2850	0.8714
RIPK2	0.0106	0.0347	0.0000	0.0000	0.1826	0.5413	1.0000	1.0000	0.1254	0.9065	0.3058	0.8714
RMC1	NA	NA	NA	NA	NA	NA	NA	NA	NA	NA	NA	NA
RNF152	0.0835	0.1695	0.3028	0.4358	0.0423	0.2442	0.1913	0.6445	0.7623	0.9719	0.3583	0.8714
RNF41	0.7254	0.8149	0.0007	0.0052	0.2559	0.6356	0.4428	0.7998	0.6866	0.9719	0.5333	0.9481

RNF5	0.1507	0.2520	0.0000	0.0007	0.6173	0.8893	0.7130	0.9350	0.6498	0.9719	0.7088	0.9709
ROCK1	0.0407	0.0952	0.7957	0.8422	0.2226	0.6145	0.9174	1.0000	0.4483	0.9719	0.4811	0.9027
RPTOR	NA	NA	0.1795	0.2956	0.2469	0.6313	0.1736	0.6371	0.0441	0.7154	NA	NA
RRAGA	0.5808	0.6991	0.5265	0.6370	0.3967	0.7637	0.0619	0.6337	0.7195	0.9719	0.4136	0.8714
RRAGB	0.0343	0.0853	0.0140	0.0465	0.0564	0.2698	0.7437	0.9350	1.0000	1.0000	0.2109	0.8714
RRAGC	0.2229	0.3295	0.7289	0.7905	0.5911	0.8860	0.2671	0.7185	0.7241	0.9719	0.3963	0.8714
RRAGD	0.9034	0.9351	0.4021	0.5529	0.0065	0.0832	0.1607	0.6371	0.8403	0.9817	0.7275	0.9709
RUBCN	NA	NA	NA	NA	NA	NA	NA	NA	NA	NA	NA	NA
RUFY4	NA	NA	NA	NA	NA	NA	NA	NA	NA	NA	1.0000	1.0000
SCFD1	0.8200	0.8697	0.2653	0.3947	0.0732	0.2992	0.7130	0.9350	0.4118	0.9719	0.1137	0.8714
SCOC	0.4793	0.6075	0.1765	0.2938	0.7849	0.9271	0.1873	0.6371	0.3358	0.9719	0.3178	0.8714
SEC22B	0.0688	0.1477	0.3390	0.4812	0.4430	0.8048	0.1261	0.6337	0.1369	0.9077	0.5010	0.9161
SESN1	0.1490	0.2509	0.0728	0.1569	0.2072	0.5932	0.0099	0.6009	0.0210	0.7154	0.2941	0.8714
SESN2	0.2001	0.3059	0.0072	0.0305	0.3223	0.7190	0.4363	0.7998	0.6139	0.9719	0.5885	0.9559
SESN3	0.7572	0.8299	0.0535	0.1268	0.6548	0.8894	0.0367	0.6009	0.4483	0.9719	0.0781	0.8714
SH3BP4	0.6077	0.7138	0.0001	0.0010	0.0273	0.1844	0.4610	0.7998	0.0441	0.7154	0.0336	0.8094
SH3GLB1	0.1651	0.2649	0.0000	0.0005	0.0389	0.2326	0.4864	0.8273	0.1254	0.9065	0.6009	0.9559
SIRT1	0.0101	0.0336	0.5582	0.6608	0.8163	0.9355	0.4363	0.7998	0.7241	0.9719	0.6904	0.9689
SIRT2	0.0009	0.0061	0.6719	0.7444	0.7803	0.9271	0.9674	1.0000	0.3358	0.9719	0.1459	0.8714
SLC38A9	NA	NA	0.1370	0.2437	0.6795	0.8894	0.3892	0.7992	0.1014	0.9025	0.2299	0.8714
SMCR8	0.0001	0.0007	0.8452	0.8736	0.7180	0.8894	0.6827	0.9217	0.2869	0.9458	0.6960	0.9709
SMG1	0.1876	0.2919	NA	NA	NA	NA	NA	NA	NA	NA	0.8014	0.9709
SNCA	0.0752	0.1550	0.1099	0.2064	0.2933	0.6824	0.0380	0.6009	0.2869	0.9458	0.3302	0.8714
SNRNP70	NA	NA	0.6167	0.7124	0.0192	0.1392	0.6827	0.9217	0.6261	0.9719	NA	NA
SNX32	NA	NA	NA	NA	0.0008	0.0562	0.0367	0.6009	0.9197	0.9942	0.8405	0.9709
SNX5	0.0000	0.0003	0.0003	0.0031	0.1445	0.4727	0.5668	0.8905	0.5553	0.9719	0.8014	0.9709

SNX6	0.9391	0.9498	0.3377	0.4812	0.5245	0.8536	0.3245	0.7266	0.0338	0.7154	0.0224	0.8094
SOGA1	NA	NA	NA	NA	NA	NA	0.4610	0.7998	0.2089	0.9077	NA	NA
SOGA3	NA	NA	NA	NA	NA	NA	NA	NA	NA	NA	NA	NA
SPTLC1	0.0028	0.0132	0.1620	0.2757	0.0052	0.0733	0.0675	0.6337	0.7623	0.9719	0.1762	0.8714
SPTLC2	0.9372	0.9498	0.0006	0.0049	0.6383	0.8894	0.2854	0.7191	0.0501	0.7154	0.2826	0.8714
SQSTM1	0.1653	0.2649	0.3765	0.5271	0.5388	0.8677	0.7130	0.9350	0.2869	0.9458	0.6148	0.9559
SREBF1	0.0581	0.1310	0.6690	0.7438	0.9681	0.9918	0.6236	0.8905	0.0577	0.7154	0.5837	0.9559
SREBF2	0.7628	0.8299	0.9038	0.9279	0.1698	0.5195	0.9674	1.0000	0.4793	0.9719	0.4970	0.9161
STAT3	0.0000	0.0000	0.0001	0.0014	0.6803	0.8894	0.7437	0.9350	0.8010	0.9719	0.0921	0.8714
STBD1	NA	NA	0.0000	0.0001	0.0544	0.2698	1.0000	1.0000	0.2642	0.9077	0.9599	0.9945
STING1	NA	NA	NA	NA	NA	NA	NA	NA	NA	NA	NA	NA
STK11	0.4796	0.6075	0.6018	0.7016	0.8941	0.9662	0.8063	0.9549	0.6139	0.9719	0.0442	0.8334
STUB1	0.8172	0.8697	0.2383	0.3745	0.1630	0.5056	0.4935	0.8313	0.2087	0.9077	0.1322	0.8714
SUPT5H	0.8191	0.8697	0.4104	0.5568	0.0883	0.3397	0.0326	0.6009	0.9795	1.0000	0.0275	0.8094
SVIP	NA	NA	0.0000	0.0001	0.0723	0.2991	0.9025	0.9941	0.3694	0.9719	NA	NA
SYNPO2	0.5113	0.6396	0.0023	0.0123	0.8374	0.9414	0.7130	0.9350	0.5114	0.9719	0.2715	0.8714
TAB2	NA	NA	0.7520	0.8042	0.4113	0.7858	0.3046	0.7266	0.1857	0.9077	NA	NA
TAB3	NA	NA	0.0120	0.0418	0.8503	0.9459	0.2496	0.7157	0.4483	0.9719	NA	NA
TBC1D14	0.0001	0.0010	0.1052	0.2013	0.1884	0.5489	0.5125	0.8388	0.2226	0.9077	0.3083	0.8714
TBC1D25	NA	NA	NA	NA	0.2388	0.6251	0.6236	0.8905	0.7241	0.9719	0.5170	0.9347
TBK1	0.0058	0.0216	0.1932	0.3165	0.1742	0.5258	0.8682	0.9933	0.0191	0.7154	0.4697	0.9027
TEX264	0.0247	0.0706	0.0460	0.1160	0.3400	0.7256	0.8381	0.9662	0.0905	0.9025	0.7275	0.9709
TFEB	0.3924	0.5272	0.1209	0.2217	0.2268	0.6145	0.6783	0.9217	0.9197	0.9942	0.0782	0.8714
TICAM1	0.6238	0.7277	0.0401	0.1041	0.6839	0.8894	0.3669	0.7810	0.0086	0.4974	0.1530	0.8714
TIGAR	NA	NA	NA	NA	NA	NA	NA	NA	NA	NA	NA	NA
TLK2	0.0686	0.1477	0.6641	0.7411	0.7067	0.8894	0.3892	0.7992	0.5788	0.9719	0.6009	0.9559

TMEM150A	NA	NA	0.0663	0.1469	0.2524	0.6356	0.3669	0.7810	0.9598	1.0000	NA	NA
TMEM150B	NA	NA	NA	NA	NA	NA	0.2854	0.7191	0.0387	0.7154	NA	NA
TMEM150C	NA	NA	NA	NA	0.0076	0.0939	0.0814	0.6337	0.0544	0.7154	NA	NA
TMEM39A	0.0339	0.0851	0.0001	0.0008	0.9739	0.9937	0.3195	0.7266	0.2035	0.9077	0.2018	0.8714
TMEM39B	0.0032	0.0142	0.0184	0.0571	0.6496	0.8894	0.2169	0.6841	0.5446	0.9719	0.0275	0.8094
TMEM59	0.0108	0.0349	0.0595	0.1343	0.8050	0.9348	0.4363	0.7998	0.3622	0.9719	0.8899	0.9786
TOMM7	0.0392	0.0931	NA	NA	NA	NA	NA	NA	NA	NA	0.9098	0.9867
TP53	NA	NA	NA	NA	0.3664	0.7439	0.1736	0.6371	0.2226	0.9077	0.3577	0.8714
TP53INP1	0.0533	0.1212	0.0519	0.1243	0.6605	0.8894	0.8063	0.9549	0.4184	0.9719	0.2299	0.8714
TP53INP2	0.3841	0.5186	0.0188	0.0578	0.4550	0.8140	0.6333	0.9007	0.1007	0.9025	0.2299	0.8714
TPCN1	0.9362	0.9498	0.1790	0.2956	0.5537	0.8740	0.8702	0.9933	0.9795	1.0000	0.2738	0.8714
TPCN2	0.0004	0.0037	0.1010	0.1969	0.5557	0.8740	0.8702	0.9933	0.5114	0.9719	1.0000	1.0000
TREM2	NA	NA	NA	NA	0.0043	0.0733	0.6529	0.9062	0.5446	0.9719	0.0559	0.8714
TRIB3	0.3777	0.5152	0.0997	0.1968	0.6583	0.8894	0.4363	0.7998	0.1389	0.9077	0.8617	0.9709
TRIM13	NA	NA	0.8070	0.8454	0.6134	0.8893	0.9674	1.0000	0.2642	0.9077	0.1605	0.8714
TRIM14	0.0000	0.0000	0.2426	0.3793	0.6186	0.8893	0.0814	0.6337	0.3107	0.9458	0.0469	0.8334
TRIM21	0.0655	0.1439	0.0029	0.0149	0.9759	0.9937	0.3245	0.7266	0.5788	0.9719	0.1411	0.8714
TRIM22	0.0000	0.0000	0.0000	0.0002	0.1160	0.4091	0.1485	0.6371	0.0120	0.5936	0.5170	0.9347
TRIM27	NA	NA	0.0002	0.0018	0.3349	0.7191	0.1607	0.6371	0.2592	0.9077	0.1762	0.8714
TRIM34	0.0695	0.1478	0.5972	0.6993	NA	NA	0.2671	0.7185	0.0338	0.7154	0.8307	0.9709
TRIM38	0.0008	0.0059	0.7100	0.7728	0.7103	0.8894	0.8381	0.9662	1.0000	1.0000	0.3921	0.8714
TRIM5	0.0000	0.0002	0.0001	0.0013	0.6366	0.8894	0.2454	0.7157	0.1857	0.9077	0.3455	0.8714
TRIM6	0.9875	0.9875	0.6603	0.7395	0.0422	0.2442	0.1844	0.6371	0.0006	0.2179	0.5541	0.9481
TRIM65	NA	NA	NA	NA	0.7887	0.9271	0.8063	0.9549	0.7976	0.9719	0.4104	0.8714
TRIM68	0.0984	0.1870	0.0597	0.1343	0.0627	0.2762	0.1873	0.6371	0.5114	0.9719	0.2524	0.8714
TRIM8	0.0067	0.0239	0.1718	0.2876	0.4524	0.8140	0.9674	1.0000	0.8575	0.9880	0.8602	0.9709

TRIML1	NA	NA	NA	NA	0.0756	0.3053	0.2671	0.7185	0.5114	0.9719	0.4208	0.8714
TRIML2	NA	NA	NA	NA	0.0040	0.0733	0.6236	0.8905	0.4184	0.9719	0.1093	0.8714
TSC1	0.1241	0.2190	0.0316	0.0846	0.8555	0.9459	0.1873	0.6371	0.7583	0.9719	0.1589	0.8714
TSC2	0.6845	0.7814	0.7429	0.7973	0.7134	0.8894	0.9349	1.0000	0.7623	0.9719	1.0000	1.0000
TSPO	0.1460	0.2473	0.0021	0.0113	0.9272	0.9803	0.0892	0.6337	0.0908	0.9025	0.8813	0.9725
UBA5	NA	NA	0.7061	0.7728	0.6888	0.8894	0.1776	0.6371	0.9197	0.9942	NA	NA
UBQLN1	0.7594	0.8299	0.6497	0.7357	0.0487	0.2550	0.1102	0.6337	0.1129	0.9065	0.9207	0.9867
UBQLN2	0.2205	0.3295	0.4225	0.5596	0.7022	0.8894	0.1102	0.6337	0.4417	0.9719	0.7842	0.9709
UBQLN4	NA	NA	NA	NA	0.0112	0.1076	0.0027	0.4688	0.5788	0.9719	0.2715	0.8714
UCHL1	0.0253	0.0714	0.4233	0.5596	0.0950	0.3475	0.3951	0.7998	1.0000	1.0000	0.1951	0.8714
UFC1	0.0100	0.0335	0.5213	0.6346	0.1061	0.3822	0.2808	0.7191	0.8175	0.9815	0.2299	0.8714
UFL1	NA	NA	0.0585	0.1343	0.1082	0.3857	0.9674	1.0000	0.7623	0.9719	NA	NA
UFM1	0.1446	0.2466	0.0006	0.0050	0.3094	0.7140	0.1261	0.6337	0.0256	0.7154	0.7088	0.9709
ULK1	0.0031	0.0139	0.1203	0.2217	0.4568	0.8140	0.4363	0.7998	0.2089	0.9077	0.2109	0.8714
USP10	0.3812	0.5174	0.0036	0.0180	0.8437	0.9452	0.2328	0.7086	0.6866	0.9719	0.2715	0.8714
USP13	0.0028	0.0132	0.1710	0.2876	0.0195	0.1392	0.2496	0.7157	0.1014	0.9025	0.2606	0.8714
USP30	0.0244	0.0706	0.0000	0.0007	0.7018	0.8894	0.7244	0.9350	0.7623	0.9719	0.3203	0.8714
USP33	0.0018	0.0099	0.4857	0.6152	0.8541	0.9459	0.7748	0.9549	0.3622	0.9719	0.6149	0.9559
USP36	0.1526	0.2537	0.5396	0.6492	0.0954	0.3475	0.2854	0.7191	0.5788	0.9719	0.1258	0.8714
UVRAG	0.8207	0.8697	0.6972	0.7697	0.8304	0.9383	0.2454	0.7157	0.7623	0.9719	0.6721	0.9559
VDAC1	0.6674	0.7653	0.0221	0.0641	0.1615	0.5056	0.1607	0.6371	0.3358	0.9719	0.4248	0.8714
VPS13C	0.2571	0.3716	0.2948	0.4303	0.9604	0.9918	0.3245	0.7266	0.4793	0.9719	0.4545	0.9027
VPS13D	0.0139	0.0435	0.8860	0.9126	0.6003	0.8860	0.4428	0.7998	0.8010	0.9719	0.7652	0.9709
VPS26A	0.0064	0.0234	0.9555	0.9618	0.5119	0.8516	0.0975	0.6337	0.7976	0.9719	0.4208	0.8714
VPS26B	0.0277	0.0761	0.0491	0.1200	0.4423	0.8048	0.8063	0.9549	0.0858	0.9025	0.9603	0.9945
VPS35	0.0869	0.1738	0.0000	0.0000	0.0604	0.2698	0.5754	0.8905	0.9598	1.0000	0.0527	0.8714

WAC	0.4353	0.5649	0.4404	0.5779	0.3173	0.7140	0.9025	0.9941	0.7241	0.9719	0.7842	0.9709
WASHC1	NA	NA	NA	NA	NA	NA	NA	NA	NA	NA	NA	NA
WDFY3	0.0003	0.0026	0.5033	0.6251	0.0334	0.2108	0.5393	0.8664	1.0000	1.0000	0.6721	0.9559
WDR24	0.3658	0.5042	0.0076	0.0317	0.5974	0.8860	0.4363	0.7998	0.3897	0.9719	0.0469	0.8334
WDR41	0.8400	0.8832	0.2100	0.3369	0.4817	0.8265	1.0000	1.0000	0.8403	0.9817	0.9405	0.9867
WDR6	0.0017	0.0097	0.8039	0.8451	0.0892	0.3397	0.9025	0.9941	0.3427	0.9719	0.0379	0.8094
WDR81	0.0026	0.0132	0.8245	0.8550	0.5655	0.8811	0.1064	0.6337	0.3107	0.9458	0.4064	0.8714
WIPI2	0.3419	0.4737	0.1474	0.2586	0.6964	0.8894	0.0465	0.6009	0.0061	0.4974	0.3557	0.8714
ZC3H12A	0.0000	0.0000	NA	NA	NA	NA	NA	NA	NA	NA	0.2265	0.8714
ZKSCAN3	NA	NA	0.1377	0.2437	0.0163	0.1366	0.0502	0.6009	0.3897	0.9719	0.6184	0.9559
ZMPSTE24	0.0369	0.0893	0.2326	0.3674	0.2496	0.6335	0.2716	0.7191	0.6866	0.9719	0.8602	0.9709
KIAA1324	0.9528	0.9564	0.0148	0.0479	0.4690	0.8224	0.3669	0.7810	0.2428	0.9077	0.4852	0.9027

Table S3. Accuracy, Precision, Recall, and F1-score of the models for Blood, CD, and UC samples.

		accuracy	precision	recall	f1-score
Blood	GLM	0.675	0.6833	0.8542	0.7593
	RF	0.6625	0.6667	0.875	0.7568
	SVM	0.6	0.6053	0.9583	0.7419
	XGB	0.7	0.7069	0.8542	0.7736
CD	GLM	0.83636	0.8571	0.9231	0.8889
	RF	0.8	0.7917	0.9744	0.8736
	SVM	0.83636	0.8846	0.8846	0.8846
	XGB	0.78182	0.814	0.8974	0.8537
UC	GLM	0.72917	0.7105	0.931	0.806
	RF	0.75	0.7742	0.8276	0.8
	SVM	0.75	0.7073	1	0.8286
	XGB	0.83333	0.8182	0.931	0.871
